# Sequential Analysis of the N/O-Glycosylation of Heavily Glycosylated HIV-1 gp120 Using EThcD-sceHCD-MS/MS

**DOI:** 10.3389/fimmu.2021.755568

**Published:** 2021-10-21

**Authors:** Yong Zhang, Shanshan Zheng, Wanjun Zhao, Yonghong Mao, Wei Cao, Wenjuan Zeng, Yueqiu Liu, Liqiang Hu, Meng Gong, Jingqiu Cheng, Younan Chen, Hao Yang

**Affiliations:** ^1^ National Health Commission (NHC) Key Laboratory of Transplant Engineering and Immunology, Institutes for Systems Genetics, National Clinical Research Center for Geriatrics, West China Hospital, Sichuan University, Chengdu, China; ^2^ Sichuan Provincial Engineering Laboratory of Pathology in Clinical Application, West China Hospital, Sichuan University, Chengdu, China; ^3^ Department of Thoracic Surgery, West China Hospital, Sichuan University, Chengdu, China; ^4^ Institute of Thoracic Oncology, West China Hospital, Sichuan University, Chengdu, China

**Keywords:** human immunodeficiency virus, envelope glycoprotein, N/O-glycosylation, EThcD-sceHCD-MS/MS, glycoproteomics

## Abstract

Deciphering the glycosylation of the viral envelope (Env) glycoprotein is critical for evaluating viral escape from the host’s immune response and developing vaccines and antiviral drugs. However, it is still challenging to precisely decode the site-specific glycosylation characteristics of the highly glycosylated Env proteins, although glycoproteomics have made significant advances in mass spectrometry techniques and data analysis tools. Here, we present a hybrid dissociation technique, EThcD-sceHCD, by combining electron transfer/higher-energy collisional dissociation (EThcD) and stepped collision energy/higher-energy collisional dissociation (sceHCD) into a sequential glycoproteomic workflow. Following this scheme, we characterized site-specific N/O-glycosylation of the human immunodeficiency virus type 1 (HIV-1) Env protein gp120. The EThcD-sceHCD method increased the number of identified glycopeptides when compared with EThcD, while producing more comprehensive fragment ions than sceHCD for site-specific glycosylation analysis, especially for accurate O-glycosite assignment. Finally, eighteen N-glycosites and five O-glycosites with attached glycans were assigned unambiguously from heavily glycosylated gp120. These results indicate that our workflow can achieve improved performance for analysis of the N/O-glycosylation of a highly glycosylated protein containing numerous potential glycosites in one process. Knowledge of the glycosylation landscape of the Env glycoprotein will be useful for understanding of HIV-1 infection and development of vaccines and drugs.

## Introduction

Human immunodeficiency virus type 1 (HIV-1) is the major cause of the life-threatening disease known as acquired immune deficiency syndrome (AIDS) (1). In particular, the HIV-1 group M viruses are more virulent than other groups and primarily responsible for the global AIDS pandemic (2). Although antiretroviral treatments have greatly improved the life of infected individuals, the development of an HIV-1 vaccine remains a top public health priority to control the HIV-1 pandemic. The envelope (Env) proteins that coat on the surface of HIV-1virions are currently the only known HIV-1-specific targets for the elicitation of broadly neutralizing antibodies (bNAbs) (3, 4). However, the Env proteins are heavily N-glycosylated and half of the mass of the proteins consists of host-derived N-glycans. This high density of glycans creates a ‘glycan shield’ that impedes antibody recognition and frequently leads to immune escape in the host cells (5). Nevertheless, approximately 10%–30% of AIDS patients can produce potent bNAbs after years of infection, and the isolated antibodies from these patients have proved to be protective against viral challenge in non-human primates and humanized mice (6–8). Intriguingly, most bNAbs recognize glycan-occupied epitopes and display glycan-dependent neutralization. Hence, the key to developing a successful HIV vaccine is the elicitation of HIV bNAbs by immunogens with native-like glycosylation patterns.

The Env glycoprotein consists of trimers of non-covalently associated gp120/gp41 heterodimers. The gp120 subunit mediates the tropism and the binding of HIV-1 to host cells, including the major target cells, CD4^+^ T cells. There are up to 24 potential N-linked glycosylation sites (PNGSs) in the gp120 subunit of most HIV-1 variants, and these PNGSs often change during virus mutation, leading to the alteration of viral infectivity (9, 10). The glycan types on the gp120 subunit play a considerable role in the immunogenicity of this Env protein. A decrease in the sialic acid content of the glycans on gp120 can increase the immunogenicity (11), while removing key glycans to unmask sites of immune vulnerability can enable the induction of bNAbs (12). Some glycan-specific bNAbs can suppress HIV-1 replication or entry into CD4^+^ cells (13). For example, 2G12, PGT121, PGT128, and PGT135 recognize the mannose-dependent epitope on gp120 (14, 15). These studies have indicated that the correct glycan types must be present in the correct sites for faithful mimicry of the gp120 glycosylation (16). Thus, a comprehensive and in-depth characterization of the glycosylation of the envelope glycoprotein gp120 is important for the rational design of vaccines and drugs targeted toward HIV-1 (17).

To achieve this characterization, researchers have been working to identify the glycosylation profiles of envelope glycoproteins produced from a variety of cell types (17, 18). The identification of the N-glycosites and some N-glycans has been performed by the analysis of simplified glycopeptides (glycosidase-treated glycopeptides) and recombinant gp120 glycoprotein. Doores et al. found that predominantly oligomannose glycans were present on the viral envelope of HIV-1 using MALDI-TOF-MS analysis (19). Wang et al. determined the glycosylation profiles of recombinant gp120 proteins from four major clades of HIV-1 using CID-MS/MS, and found that over 40% of the glycans present on gp120 were high-mannose glycans (18). Struwe et al. assessed the global occupancy of glycosites by intact mass spectrometry after simplifying the spectra of the HIV-1 gp120 using glycan metabolic engineering to homogenize the processing of N-linked glycans and eliminate glycan heterogeneity (20). Cao et al. sequentially treated peptides with two specific endoglycosidases End H and PNGase F to determine the degree of glycan occupancy and the proportion of high-mannose and complex-type glycans at each glycosite of the HIV-1 Env using CID-MS/MS (21, 22). However, these studies mainly focused on N-glycans, N-glycosites, and N-glycan site occupancy. The in-depth characterization of site-specific glycosylation profile calls for detection at the intact glycopeptide level, which is fundamental for glycopathology analysis and precision theranostics.

The analysis of intact glycopeptides is challenging because of the glycan microheterogeneity, the low proportion of glycopeptides in digested samples, and the ion suppression effects of unmodified peptides (23–25). Even so, some studies have attempted to characterize the intact glycopeptides of gp120, which can reflect the N-glycan synthesis that starts with high-mannose glycans and further processes into hybrid and complex N-glycans in the Golgi apparatus (26). Panico et al. have used the intact glycopeptides and deglycosylated peptides to systematically profile the site-specific N-glycosylation of gp120 derived from virions by using LC-ESI (electrospray ionization) MS and LC-MALDI-TOF MS (27). Behrens et al. analyzed the glycopeptides from BG505 SOSIP.664 trimers using both MALDI-TOF MS and HCD-MS/MS (28). GO et al. have characterized Env N-glycosylation using CID-、HCD- or ETD-MS/MS (29–33). These studies have used different ionization modes and different fragmentation techniques (34). However, the in-depth N-glycosylation analysis needs to improve both the number of identified N-glycopeptides and the accurate determination of the glycosite-specific occupancy by different glycoforms. Compared with N-glycosylation analysis, the profiling of intact O-glycopeptides is more challenging because of the lack of a defined O-glycosylation motif and an enzyme that can remove all the O-glycans for site-specific characterization (35). Currently, the biological function of O-glycosylation on the viral Env protein is poorly understood (36, 37). To date, there have been limited reports regarding the in-depth analysis of both N- and O-glycosylation of heavily glycosylated viral Env proteins through sequential analysis of the N/O-glycopeptides.

In this work, we present a sequential glycoproteomic workflow for the characterization of the intact N/O-glycopeptides of the HIV-1 Env gp120 based on two MS methods, sceHCD-MS/MS and EThcD-sceHCD-MS/MS. We have obtained the in-depth and high accurate N/O-glycosylation profile for the heavily glycosylated protein gp120. The N-glycosylation profile of gp120 contained high mannose-, complex- and hybrid-type glycans at each N-glycosite because of the microheterogeneity. The O-glycosylation profile of gp120 revealed some unreported O-glycosites and the O-glycans at these sites. This method for obtaining detailed and high-quality information regarding the N/O-glycosylation of gp120 will assist our understanding of N/O-glycosylation on highly glycosylated proteins from different types of viruses, such as HIV, SARS-CoV, and SARS-CoV-2 (38). Such information will also provide guidance on how to assess immunogens with optimal glycosylation for vaccine development.

## Experimental Procedures

### Experimental Design and Statistical Rationale

Recombinant HIV-1 gp120 protein (100 μg) expressed in human cells was digested using trypsin and a combination of trypsin and Glu-C. The digestion products were enriched by hydrophilic interaction liquid chromatography (HILIC) and digested using PNGase F. Finally, the intact N-glycopeptides before and after enrichment and the deglycosylated peptides were analyzed by sceHCD-MS/MS and EThcD-sceHCD-MS/MS. Data were analyzed using Byonic software (version 3.6.0, Protein Metrics, Inc.) and verified manually. Three technical replicates were used. The number of intact N-glycopeptides and N-glycans identified from triplicates was analyzed using Student’s *t*-test for statistical comparison between two groups. Data are presented as the mean ± SD, and statistical significance was set at *P* < 0.05.

### Materials

Dithiothreitol (DTT), iodoacetamide (IAA), formic acid (FA), trifluoroacetic acid (TFA), tris(hydroxymethyl)aminomethane (TRIS), and urea were purchased from Sigma (St. Louis, MO, USA). Acetonitrile (ACN) was purchased from Merck (Darmstadt, Germany). HILIC materials were obtained from Agela Technologies (Tianjin, China). Commercially available recombinant HIV-1gp120 protein (group M, subtype B, isolate BAL, His tag) expressed in human embryonic kidney cells (HEK293) was purchased from Sino Biological (Beijing, China). Sequencing-grade trypsin and Glu-C were obtained from Enzyme & Spectrum (Beijing, China). The quantitative colorimetric peptide assay kit was purchased from Thermo Fisher Scientific (Waltham, MA, USA). Deionized water was prepared using a Milli-Q system (Millipore, Bedford, MA, USA). All other chemicals and reagents were of the best available grade and were purchased from Sigma-Aldrich or Thermo Fisher Scientific.

### Protein Digestion

Recombinant HIV-1 gp120 protein was proteolyzed using a filter-aided sample preparation (FASP) digestion protocol. Briefly, 50 μg of protein in a tube was diluted with 100 μL of 50 mM NH_4_CO_3_ and denatured for 10 min at 95°C. After reduction by DTT (20 mM) for 45 min at 56°C and alkylation with IAA (50 mM) for 1 h at 25°C in the dark, the mixture was transferred to a 30-kDa filter tube. After centrifugation at 13,000 × *g* for 15 min, the protein was washed twice with 200 μL of 50 mM NH_4_CO_3_. Then, 2 μg of trypsin or trypsin/Glu-C (w:w=1:1) was added to the filter and incubated for 4 h at 37°C. In NH_4_CO_3_ buffer, Glu-C will preferentially cleave at glutamic acid residue. The peptides were obtained by washing twice with 100 μL of water and centrifuging at 13,000 × *g* for 15 min. The peptide concentration was determined using a peptide assay kit. The peptide mixtures (intact N-glycopeptides before enrichment) were freeze-dried for further analysis.

### Enrichment of Intact N-Glycopeptides

Intact N-glycopeptides were enriched using HILIC (Agela Technologies, Tianjin, China). Specifically, 10 μg of peptides were resuspended in 100 μL of 70% ACN/0.2% TFA solution. HILIC (10 mg) was washed three times for 10 min each with 0.1% TFA and 80% ACN/0.2% TFA. Activated HILIC material (1 mg) was added to the peptide solution and incubated for 2 h at 37°C. Finally, the mixture was transferred to a 200 μL pipette tip packed with a C8 membrane and washed twice with 70% ACN/0.2% TFA. After enrichment, the intact N-glycopeptides were eluted three times with 70 μL of 0.1% TFA and dried using a SpeedVac for further analysis.

### De-N-Glycosylation

Enriched intact N-glycopeptides were digested using 2 U PNGase F dissolved in 50 μL of 50 mM NH_4_HCO_3_ for 2 h at 37°C. The reaction was terminated by the addition of 0.1% FA.

### Liquid Chromatography-Tandem Mass Spectrometry Analysis

All samples were analyzed using an Orbitrap Fusion Lumos mass spectrometer (Thermo Fisher Scientific). In brief, intact N-glycopeptides before and after enrichment and deglycosylated peptides were dissolved in 0.1% FA and separated on a column (ReproSil-Pur C18-AQ, 1.9 μm, 75 μm inner diameter, length 20 cm; Dr. Maisch) over a 78 min gradient (buffer A, 0.1% FA in water; buffer B, 0.1% FA in 80% ACN) at a flow rate of 300 nL/min.

First, each sample was subjected to sceHCD-MS/MS. The parameters were as follows: MS1 was analyzed using a scan range (m/z) of 800–2000 (intact N-glycopeptides before and after enrichment) or 350–1550 (deglycosylated peptides) at an Orbitrap resolution of 120,000. The RF lens, AGC target, maximum injection time, exclusion duration, and cycle time were 30%, 2.0 e^4^, 100 ms, 15 s, and 3 s, respectively. The precursor ion in MS2 experiment was selected with an isolation window of 2 m/z and acquired in Orbitrap mass analyzer at a resolution of 15,000. The AGC target, maximum injection time, and HCD collision energy were custom, 250 ms, and 30%, respectively. The stepped collision mode was turned on with an energy difference of ±10%.

Second, the same sample was subjected to EThcD-MS/MS. The parameters were as follows: MS1 was analyzed using a scan range (m/z) of 800–2000 at an Orbitrap resolution of 60,000. The RF lens, AGC target, maximum injection time, exclusion duration, cycle time, and intensity threshold were 30%, 4.0 e5, 50 ms, 15 s, 3 s, and 5.0 e4, respectively. The precursor ion in MS2 experiment was performed with an isolation window of 2 m/z and acquired in Orbitrap mass analyzer at a resolution of 30,000. The first mass was fixed at m/z = 120. The AGC target, maximum injection time, and EThcD collision energy were 4.0 e5, 150%, and 35%, respectively.

Third, the same samples were subjected to EThcD-sceHCD-MS/MS. Data acquisition for the EThcD-sceHCD-MS/MS was performed using an alternative fragmentation between the EThcD and sceHCD modes in a duty cycle (**Supplementary Figure S1**). This combined fragmentation strategy capitalized on the advantages of both EThcD and sceHCD to produce more comprehensive fragment ions for site-specific glycosylation analysis. Description of the detailed parameters is as follows: In duty cycle 1 (EThcD), MS1 was analyzed using a scan range (m/z) of 400–1600 at an Orbitrap resolution of 60,000. The RF lens, AGC target, maximum injection time, exclusion duration time, and cycle time were 30%, custom, 50 ms, 15 s, and 2s, respectively. The precursor ion in MS2 experiment was selected with an isolation window of 2 m/z and acquired in Orbitrap mass analyzer at a resolution of 30,000. The AGC target, maximum injection time, and EThcD collision energy were custom, 150 ms, and 35%, respectively. In duty cycle 2 (sceHCD), MS1 was analyzed using a scan range (m/z) of 400–1600 at an Orbitrap resolution of 60,000. The RF lens, AGC target, maximum injection time, exclusion duration, and cycle time were 30%, standard, auto, 15 s, and 1 s, respectively. The precursor ion in MS2 experiment was selected with an isolation window of 1.6 m/z and acquired in Orbitrap mass analyzer at a resolution of 30,000. The AGC target, maximum injection time, and HCD collision energy were 200%, auto, and 30%, respectively. The stepped collision mode was turned on with an energy difference of ±10%.

### Data Analysis

The raw data files were examined against the recombinant HIV-1 gp120 protein sequence using Byonic software (version 3.10.10, Protein Metrics, Inc.) with the mass tolerance for precursors and fragment ions set at ±10 ppm and ±20 ppm, respectively. Two missed cleavage sites were allowed for trypsin (cleavage at K and R) and/or Glu-C (preferential cleavage at E) digestion. HCD or EThcD or EThcD-HCD was chosen as the fragmentation type. The fixed modification was carbamidomethyl (C), and variable modifications included oxidation (M), acetyl (protein N-term), and deamidation (N). In addition, 182 human N-glycans were specified as N-glycan modifications for intact N-glycopeptides before and after enrichment. Six human O-glycans were specified as O-glycan modifications for de-N-glycosylated intact O-glycopeptides. Subsequently, the protein database options were selected, including the decoy database. All other parameters were set at default values, and protein groups were filtered to a 1% false discovery rate based on the number of hits obtained for searches against these databases. Strict quality control methods for intact glycopeptides and peptide identification were implemented, requiring a score of >200 and identification of at least six amino acids. Furthermore, all glycopeptide-spectrum matches (GPSMs) were manually examined. The N-glycan compositions were divided into four types (i.e., high-mannose, hybrid, complex, and others). Different types and numbers of glycan compositions on each glycosite of the recombinant HIV-1 gp120 protein were counted based on the number of their glycopeptide spectra. The relative abundance is the ratio of the number of each glycan type to the total, and the size of the pies represents the number of glycan compositions.

## Results and Discussion

The HIV-1 envelope glycoprotein gp120 plays a critical role in inducing glycan-dependent HIV neutralizing antibodies and protective immunity (10, 39). Previous studies have profiled N-glycans released from gp120 by MALDI-MS/MS and the corresponding N-glycosites by LC-MS/MS (28). The simplified N-glycopeptides resulting from intact N-glycopeptides by treatment with Endo H or PNGase F were also analyzed by LC-MS/MS to profile the N-glycan site occupancy of gp120 (18, 30). However, the analysis of intact N/O-glycopeptides of HIV-1 gp120 has rarely been systematically performed using appropriate methods. This is because sequential and in-depth analysis of the N/O-glycosylation of a highly glycosylated protein containing many potential glycosites is very challenging and time-consuming. However, characterization of the N/O-glycan composition and structure for each glycosite of HIV-1 gp120 would be useful for the development of vaccines and targeted drugs. Herein, we aimed to decode the detailed N/O-glycosylation profile of HIV-1 gp120 with high accuracy.

**Figure 1:**
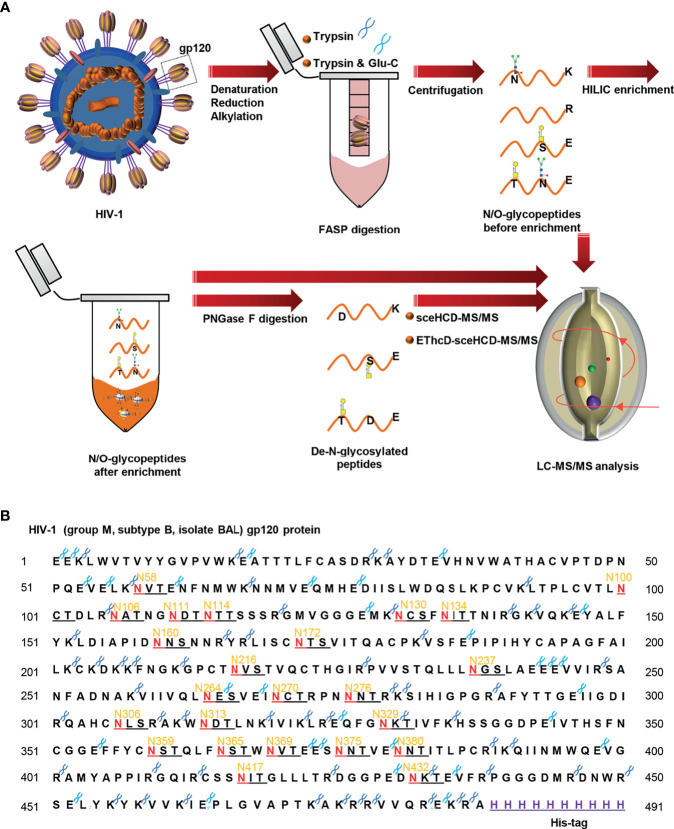
Workflow for sequential N/O-glycosylation characterization of recombinant HIV-1 gp120 protein using two complementary proteases for digestion and an integrated glycoproteomic analysis using sceHCD-MS/MS and EThcD-sceHCD-MS/MS. **(A)** Overall experimental schematic diagram. **(B)** The amino acid sequence of HIV-1 gp120 protein and potential N-glycosites and enzyme cleavage sites. The dark blue and light blue “scissors” represent the potential digestion sites of trypsin and Glu-C respectively. The red letter “N” represents the potential N-linked glycosylation site (PNGS).

To characterize the sequential N/O-glycosylation of gp120, an integrated N/O-glycoproteomic workflow using sceHCD-MS/MS and EThcD-sceHCD-MS/MS composed of the following steps was developed (**Figure 1A**) (1). The gp120 subunit was denatured, reduced, alkylated, and transferred to a 30-kDa filter tube (2). The protein was digested into peptides using trypsin or trypsin/Glu-C, according to a theoretical analysis of the enzyme cutting site. Intact N/O-glycopeptides without enrichment were analyzed by sceHCD-MS/MS and EThcD-sceHCD-MS/MS (3). The remaining intact N/O-glycopeptides were enriched by HILIC and analyzed by sceHCD-MS/MS and EThcD-sceHCD-MS/MS (4). Alternatively, these enriched intact N/O-glycopeptides were digested using PNGase F to remove N-glycans. The de-N-glycopeptides were then analyzed using the same MS method. Using this integrated method, the intact N/O-glycopeptides of a highly glycosylated protein containing many potential glycosites can be analyzed in a single process. Whole or truncated gp120 and even glycopeptide fragments can be engineered and have been used in previous studies (8). Recombinant HIV-1gp120 protein (group M, subtype B, isolate BAL, His tag) expressed in human embryonic kidney cells (HEK293) was employed in this study (**Figure 1B**). The gp120 protomer contains 24 PNGSs, which fit the consensus motif (N-X-S/T, X≠P) for N-linked glycosylation, and some serine and tyrosine residues might be O-glycosylated.

To generate high-quality MS/MS spectra appropriate for glycopeptides from HIV-1 gp120, an integrated method using sceHCD-MS/MS and EThcD-sceHCD-MS/MS was developed (**Figure 1**). The reasons for using this method include the following: (1) previous studies have focused on understanding the glycan occupancy of individual PNGS (10, 18, 30). Hence, sample preparation workflows usually exclude an intact glycopeptide enrichment step to prevent the loss of non-glycopeptides. Without an enrichment step, many N/O-glycopeptides at medium and low abundances could not be identified because of the signal inhibition from the non-glycopeptide matrix. Thus, the number of intact glycopeptides identified was limited (40, 41). Therefore, we integrated intact glycopeptides before and after enrichment into an analytical strategy to achieve a sequential analysis of the intact N/O-glycopeptides of gp120. (2) CID, HCD, sceHCD, ETD, HCD-product-dependent (pd)-ETD, ETciD, and EThcD have emerged as potentially suitable mass spectrometry methods for the analysis of intact glycopeptides (35, 42–44). For example, individual sceHCD or EThcD has been used for comprehensive N/O-glycosylation analysis of SARS-CoV-2 spike proteins (43, 45). We integrated these two superior fragmentation modes (EThcD and sceHCD) into one method (EThcD-sceHCD-MS/MS) and compared it with other methods (sceHCD-MS/MS and EThcD-MS/MS) for the highly accurate analysis of HIV-1 gp120 intact N/O-glycopeptides for the first time.

**Figure 2:**
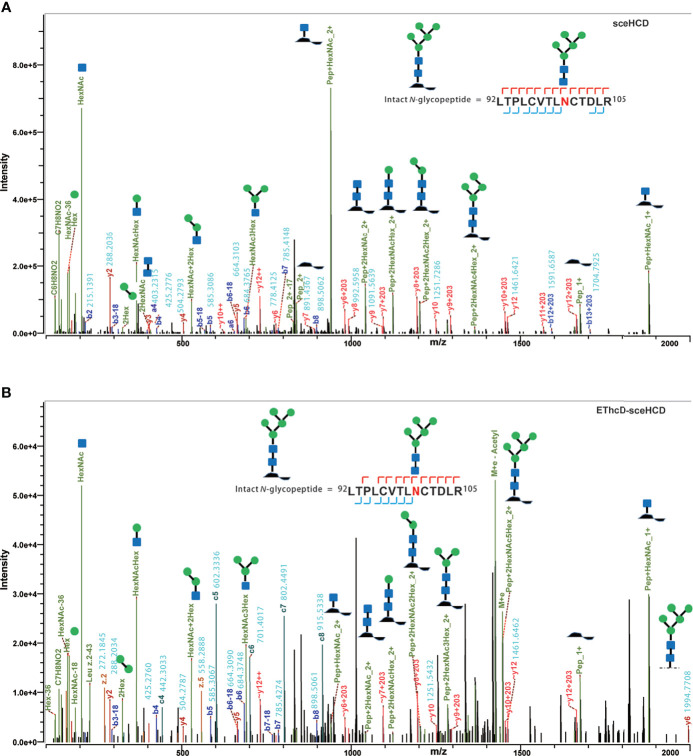
Representative sceHCD-MS/MS **(A)** and EThcd-sceHCD-MS/MS **(B)** spectra of an intact N-glycopeptide with a high mannose type N-glycan, HexNAc2Hex5, at amino acid position 100.

**Figure 3:**
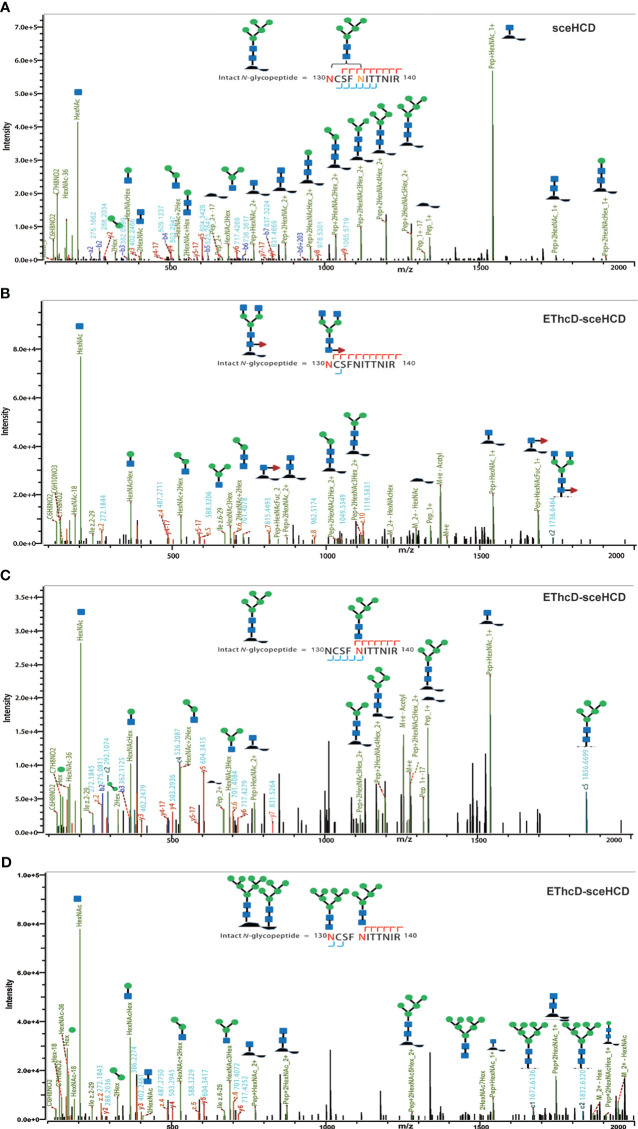
Representative sceHCD-MS/MS **(A)** and EThcd-sceHCD-MS/MS **(B–D)** spectra of intact N-glycopeptides with two potential N-glycosites (N130 and N134).

It is important to use an appropriate fragmentation mode to map the exact N-linked glycosylation of HIV-1 gp120. Previous reports have suggested that CID-, ETD-, ETciD-, HCD-pd-ETD-, and EThcD-MS/MS generated fewer fragment ions than HCD-MS/MS. SceHCD-ms/MS has been shown to generate the most informative and abundant fragment ions for both the glycan and peptide of an intact N-glycopeptide in a single spectrum (46). However, sceHCD-MS/MS cannot provide spectral evidence for the accurate location of N-glycosites when multiple N-glycosites occur in the same glycopeptide. In addition, sceHCD is also inadequate for intact O-glycopeptide analysis because one O-glycopeptide often contains more than one potential O-glycosite. EThcD can be used to fragment parent ions *via* ETD in the ion trap, and subsequently, the precursors and product ions can be transferred to an HCD collision cell for further fragmentation. This method can provide a more complete fragmentation of glycopeptides than HCD or ETD alone and allows glycosites to be unambiguously determined with a greater proportion of the fragment ions observed (46). However, the dissociation efficiency of EThcD is still limited, especially for low-charge-density precursors such as glycopeptides. Hence, we propose that EThcD-sceHCD-MS/MS can be used complementarily to provide sufficient dissociation efficiency and higher spectral quality because it can produce both sceHCD and EThcD spectra. As shown in **Figure 2**, both sceHCD-MS/MS and EThcD-sceHCD-MS/MS provided accurate N-glycosite location data and abundant information concerning the N-glycan composition. The intact N-glycopeptide (^92^LTPLCVTLNCTDLR^105^) contained a high-mannose type N-glycan, HexNAc2Hex5, at N100. The sceHCD-MS/MS spectrum contained b/y-type peptide backbone fragments, oxonium ions, and Y ions (y-type peptide backbone + glycan fragments). This peptide tended to lose part of the N-glycan during activation without the intact glycan (HexNAc2Hex5) (**Figure 2A**). Representative spectra of intact N-glycopeptides with unambiguously assigned N-glycosites using sceHCD-MS/MS are shown in **Supplementary Figure S2**. In addition to the assignment of Byonic software, these spectra contain enough manually examined ions to identify these N-glycosites accurately. In contrast, the EThcD-sceHCD-MS/MS spectrum contained b/y-type peptide backbone fragments, c/z-type peptide backbone fragments that retained intact glycan moieties with few glycan dissociation events, oxonium ions, and Y ions. The spectrum did not show a divalent intact N-glycopeptide ion or a Y ion (y6+HexNAc2Hex5) that contained the intact N-glycan HexNAc2Hex5. EThcD-sceHCD-MS/MS tended to retain intact N-glycans and provide more fragment ions during the activation process than sceHCD-MS/MS (**Figure 2B**). Representative spectra of intact N-glycopeptides with unambiguously assigned N-glycosites using EThcD-sceHCD-MS/MS are shown in **Supplementary Figure S3**. Hence, compared with sceHCD-MS/MS, using EThcD-sceHCD-MS/MS can increase confidence in the identified N-glycan. For the spectra of intact N-glycopeptides with two potential N-glycosites (N130 and N134), sceHCD-MS/MS could only be used to assign N-glycosites ambiguously (**Figure 3A**). However, EThcD-sceHCD-MS/MS can provide sufficient site-specific information (especially for c/z ions containing peptide backbone and glycan fragments) to assign these N-glycosites unambiguously. For example, the intact N-glycopeptide, ^130^NCSFNITTNIR^140^, was determined to be N-glycosylated at N130, mainly based on the c2 ion (c-type peptide backbone fragment + HexNAc4Hex3Fuc1) and z7-z10 ions without additional glycan fragments (**Figure 3B**). The same N-glycopeptide can also be N-glycosylated at N134 based on the presence of the c5 ion (c-type peptide backbone fragment + HexNAc2Hex5) and c2/c4 ions lacking glycan moieties (**Figure 3C**). **Figure 3D** shows that the N-glycopeptide can be N-glycosylated at N130 and N134 based on the delta mass of the observed intact glycopeptides (M+H, 4096.6016 Da) compared with the calculated de-N-glycopeptide (M+H, 1339.6423 Da). Further assignment of the glycans to specific sites resulted from the observed c1 (c-type peptide backbone fragment + HexNAc2Hex7), c2 (c-type peptide backbone fragment + HexNAc2Hex7), Y (y-type peptide backbone + HexNAc2Hex5), and other ions, which indicated that N130 was modified by HexNAc2Hex7 (1540.5285 Da), implying that N134 was modified by HexNAc2Hex5 (1216.4229 Da). In addition, EThcD can also increase the confidence of the identified N-glycans and N-glycosites. However, both sceHCD and EThcD-sceHCD outperformed EThcD in terms of identification (**Supplementary Figure S4**). Therefore, compared with sceHCD-MS/MS and EThcD-MS/MS, EThcD-sceHCD-MS/MS can increase the identification of intact N-glycopeptides and confidence in both the identified N-glycans and N-glycosites.

**Figure 4:**
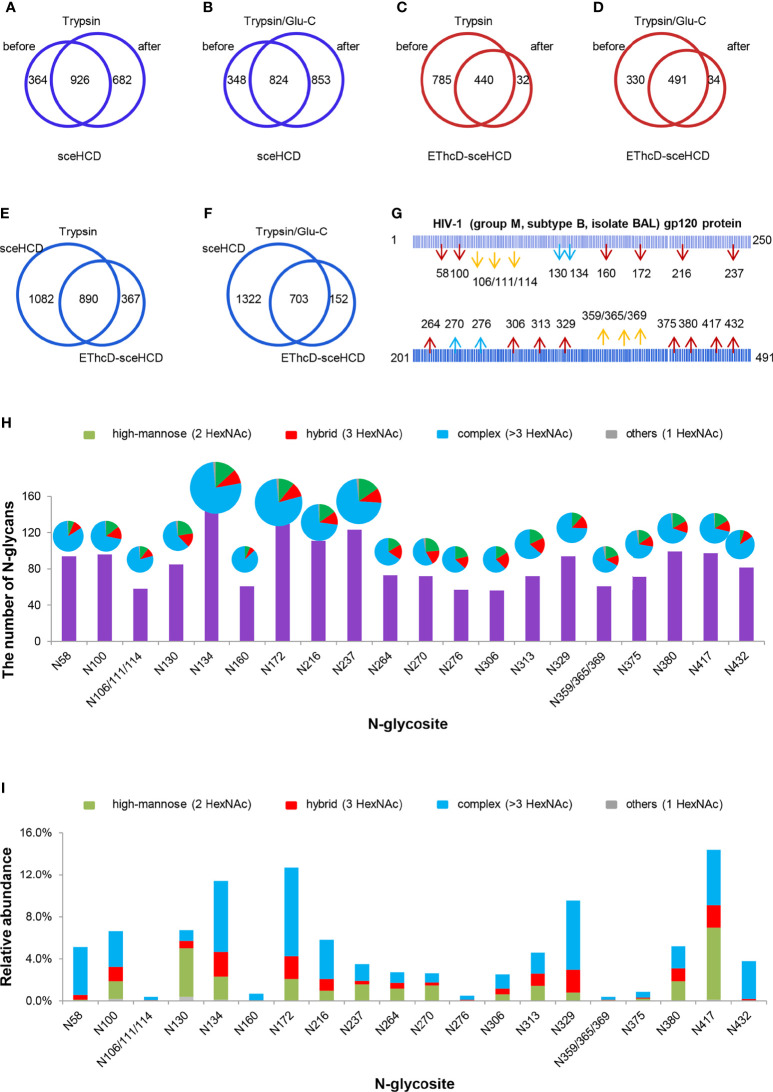
Site-specific N-glycosylation of HIV-1 gp120 protein. **(A, B)** Comparison of the number of trypsin **(A)** or trypsin/Glu-C **(B)** digested intact N-glycopeptides identified by sceHCD-MS/MS before and after enrichment. **(C, D)** Comparison of the number of trypsin **(C)** or trypsin/Glu-C **(D)** digested intact N-glycopeptides identified by EThcD-sceHCD-MS/MS before and after enrichment. **(E, F)** Comparison of the number of trypsin **(E)** or trypsin/Glu-C **(F)** digested intact N-glycopeptides identified by sceHCD-MS/MS and EThcD-sceHCD-MS/MS. **(G)** Glycoproteomic identification of the glycosites on recombinant HIV-1 gp120 protein. The red arrows indicate that the glycosite was unambiguously assigned by both sceHCD-MS/MS and EThcD-sceHCD-MS/MS. The blue arrows indicate that the glycosite was only unambiguously assigned by EThcD-sceHCD-MS/MS. The yellow arrows indicate that the glycosite was ambiguously assigned by sceHCD-MS/MS or EThcD-sceHCD-MS/MS. **(H)** Different types and numbers of N-glycans at each N-glycosite of the recombinant HIV-1 gp120 protein. The size of the pies represents the variety of N-glycan compositions. **(I)** The ratio of number of spectra of the four N-glycan types on individual N-glycosites to the number of spectra of all the N-glycosites.

**Figure 5:**
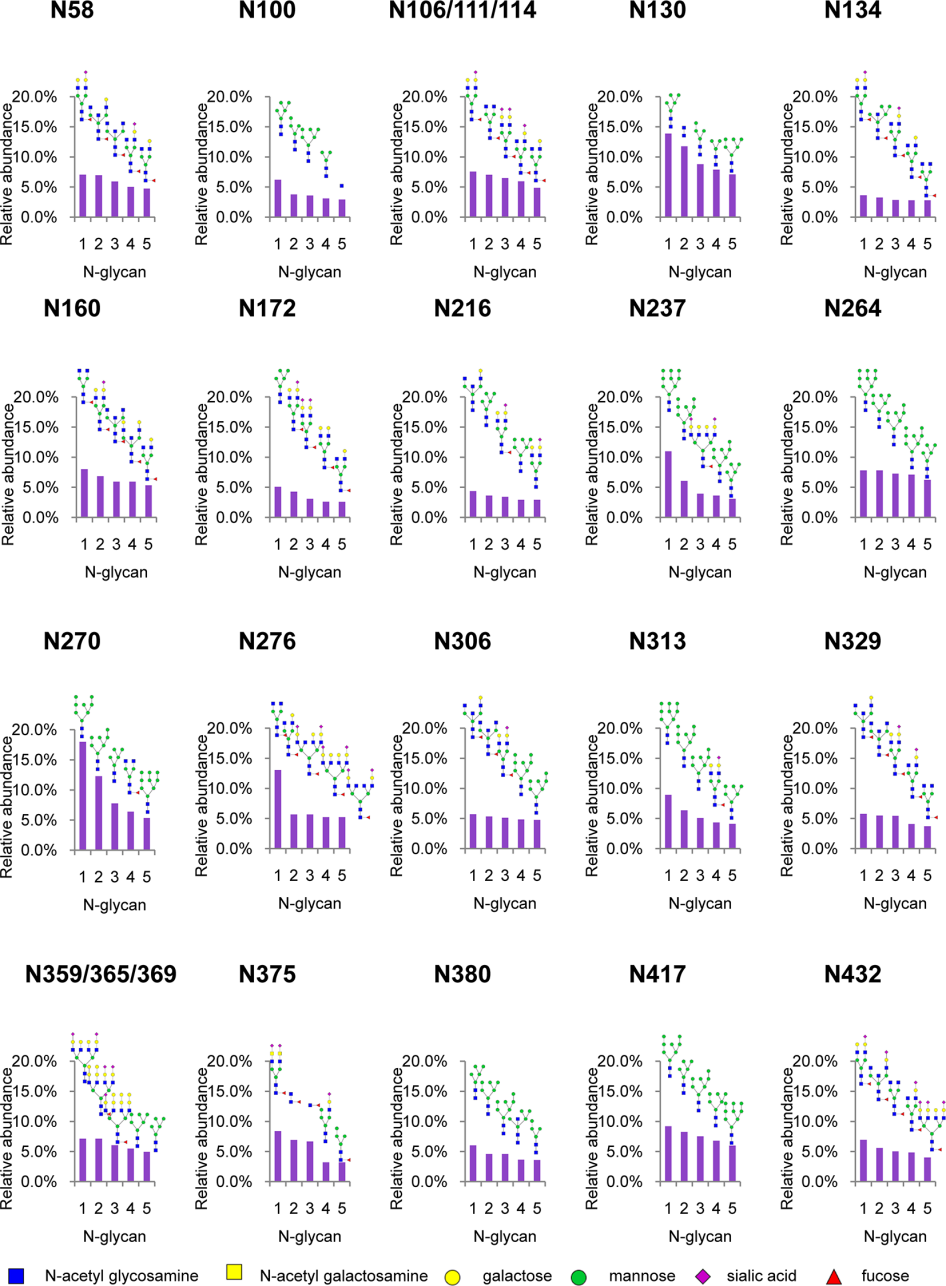
Relative abundance of the top five N-glycans on individual glycosites of recombinant HIV-1 gp120 protein. The data represent the relative ratio of each N-glycans on one glycosite. Three replicates were used for the final statistical analysis.

To analyze intact N/O-glycopeptide data, there are some excellent software products available, including pGlyco2.0 (47), glycobinder (48), GPQuest (49), Byonic (50), StrucGP (51) and MSFragger-Glyco (52). We used Byonic software to analyze data from intact glycopeptides produced by sceHCD-MS/MS, EThcD-MS/MS, and EThcD-sceHCD-MS/MS. More trypsin-or trypsin/Glu-C-digested intact N-glycopeptides from recombinant HIV-1 gp120 protein could be identified by sceHCD-MS/MS after enrichment using HILIC (**Figures 4A, B**) compared with using EThcD-sceHCD-MS/MS after enrichment using HILIC (**Figures 4C, D**). These results indicated that EThcD-sceHCD scans were slower than sceHCD scans, resulting in fewer MS/MS acquisitions. Moreover, sceHCD-MS/MS could be used to identify more intact N-glycopeptides compared to using EThcD-sceHCD-MS/MS. That is, EThcD-sceHCD-MS/MS sacrificed the number of intact glycopeptides identified for fragmentation quality. Approximately half of the trypsin-or trypsin/Glu-C-digested intact N-glycopeptides could be determined by both sceHCD-MS/MS and EThcD-sceHCD-MS/MS (**Figures 4E, F**). Of the 24 PNGSs on the gp120, 14 glycosites (N58, N100, N160, N172, N216, N237, N264, N306, N313, N329, N375, N380, N417, and N432) were unambiguously assigned by both sceHCD-MS/MS and EThcD-sceHCD-MS/MS. Four glycosites (N130, N134, N270, and N276) were unambiguously assigned by EThcD-sceHCD-MS/MS, and six glycosites (N106, N111, N114, N359, N365, and N369) were ambiguously assigned by sceHCD-MS/MS or EThcD-sceHCD-MS/MS (**Figure 4G**, **Supplementary Figures S2**, **S3** and **Supplementary Table S1**). Detailed information on the intact N-glycopeptides and glycosites is shown in **Supplementary Tables S2** and **S3**. Nearly all the N-glycosites contained over 50 types of N-glycans, more than half of which were complex-type glycans, followed by high-mannose type and hybrid type glycans (**Figure 4H** and **Supplementary Table S4**), and the number of identified N-glycans was far more than has been previously reported (10, 18). It is worth noting that the N-glycosites N106/111/114 and N359/365/369 were located in one glycopeptide. Hence, site-specific N-glycosylation information could not be provided (**Supplementary Table S4**). There are four N-glycosites (N134, N172, N216, and N237) that were decorated with markedly heterogeneous N-glycans of up to 100 different types. The relative abundances of the different types of N-glycans on each N-glycosite of gp120 are shown in **Figure 4I** and **Supplementary Table S5**. The results indicated that the relative abundances of high mannose-type, complex-type, and hybrid-type glycans were 27.8%, 16.9%, and 54.2%, respectively. In addition, four N-glycosites (N130, N264, N270, and N417) were mainly decorated with high mannose-type N-glycans (over 50%), suggesting that little processing occurred at these sites, which correlated with the expected “mannose patch.” Other sites were mainly decorated with complex-type N-glycans, implying that most N-glycosites were heavily processed. Furthermore, we determined the relative abundance of the top five N-glycans on the individual glycosites of gp120. Intriguingly, high mannose-type N-glycans were the N-glycans with the highest abundance in nine N-glycosites (N100, N130, N172, N237, N264, N270, N313, N380, and N417), although high mannose-type glycan types comprised a very low proportion of the total N-glycans (**Figure 5** and **Supplementary Table S5**). Previous studies have also shown that monomeric gp120 carries a range of highly processed complex-type glycans (~70%) together with a smaller population of unprocessed oligomannose-type glycans (~30%), while trimeric gp120 or gp120 isolated from the primary virus usually have a high abundance of oligomannose glycans (53, 54). Go et al. found that the transmitted/founder (TF) Env was more heavily mannosylated than the Env of chronic infection (CI) variants (31, 55). It is worth noting that the composition and abundance of N-glycans are relevant to immunogen design because certain complex-type glycans can also contribute to bNAb epitopes and immunogenicity (30, 56, 57).

**Figure 6:**
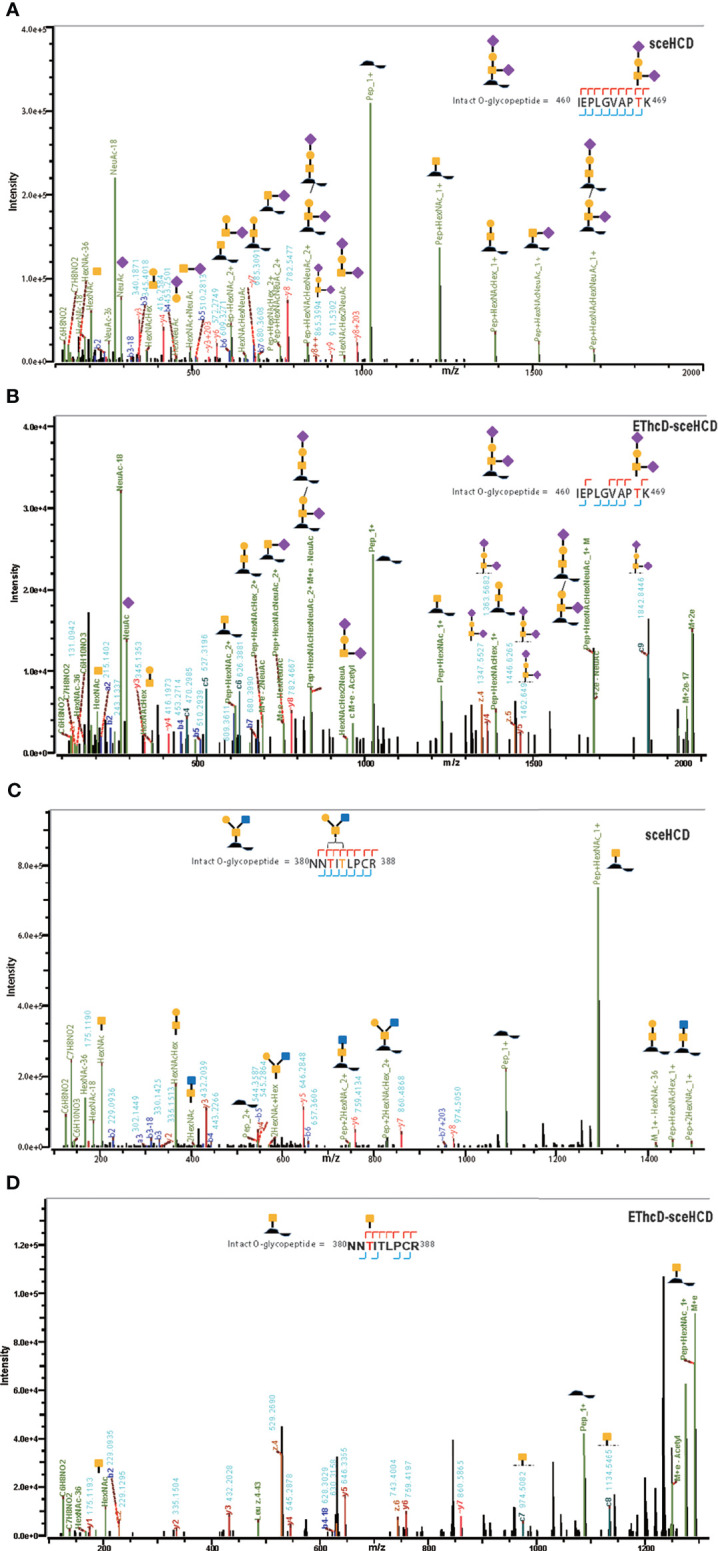
Representative sceHCD-MS/MS **(A, C)** and EThcd-sceHCD-MS/MS **(B, D)** spectra of intact O-glycopeptides with one (T468) or two (T382 and T384) potential O-glycosites.

Although gp120 proteins are known to have very few O-glycosites, there is a lack of systematic and in-depth studies on the O-glycosylation of gp120 (16). In previous research, the number of identified O-glycoPSMs was lower than is desired to draw conclusions (46, 58). In this study, an integrated workflow was used for intact O-glycopeptide identification (**Supplementary Tables S6**, **S7**). While some O-glycan-retaining fragments have been detected in HCD and sceHCD spectra, they are often not sufficient for determining the glycosite location in intact O-glycopeptides because of the multiple serine and/or threonine residues, which can lead to ambiguity (46). Fortunately, a few O-glycosites can be determined because of the presence of only one potential O-glycosite in an intact O-glycopeptide of gp120. However, the percentage of O-glycoPSMs was low. For example, the intact O-glycopeptide (^460^IEPLGVAPTK^469^) contains the O-glycan HexNAc2HexNeuAc2 at T468. Both the sceHCD-MS/MS and EThcD-sceHCD-MS/MS spectra can provide sufficient ion information to assign only O-glycosite (T468) and O-glycan (HexNAc2HexNeuAc2) with confidence (**Figures 6A, B**). Compared with the sceHCD-MS/MS spectrum, the EThcD-sceHCD-MS/MS spectrum contained c/z-type ions and more Y ions that retained the intact O-glycan. This information will help to unambiguously assign O-glycosites and O-glycans. In addition, the intact O-glycopeptide (^380^NNTITLPCR^388^) contains two potential O-glycosites (T382 and T384). From the sceHCD-MS/MS spectrum (**Figure 6C**), we can determine the O-glycan composition (HexNAc2Hex1), but not the O-glycosite (T382 or T384). However, using the EThcD-sceHCD-MS/MS spectrum (**Figure 6D**), we can determine both the O-glycan composition (HexNAc1) and the O-glycosite (T382) mainly because of the presence of key ions, such as z6, c7, and c8. Representative spectra of intact O-glycopeptides with unambiguously assigned O-glycosites using sceHCD-MS/MS and EThcD-sceHCD-MS/MS are shown in **Supplementary Figures S5**, **S6**, respectively. Hence, our data showed that EThcD-sceHCD-MS/MS was generally more reliable at generating fragment ion types sufficient for the robust characterization of intact O-glycopeptides compared with sceHCD-MS/MS.

**Figure 7:**
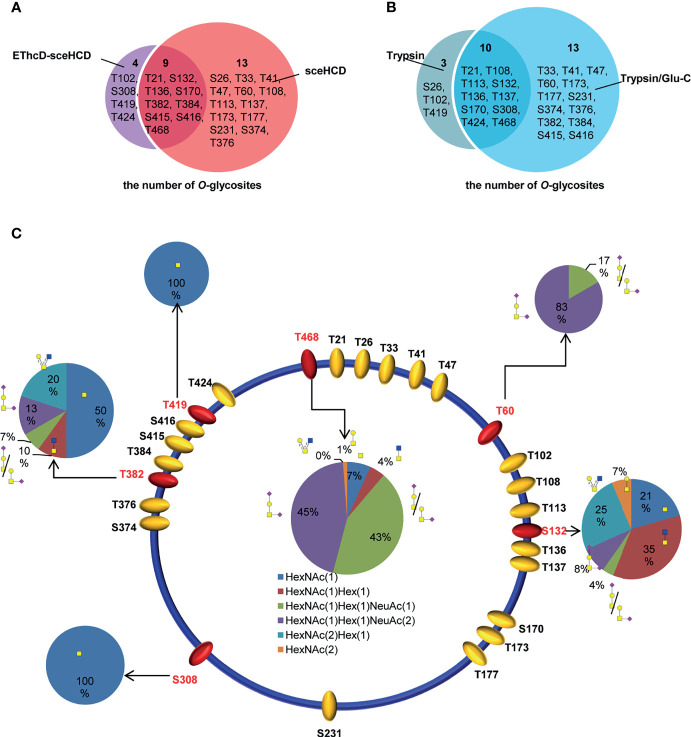
Site-specific O-glycosylation of HIV-1 gp120 protein. **(A)** The number of O-glycans in gp120 identified using sceHCD-MS/MS and EThcD-sceHCD-MS/MS. **(B)** The number of O-glycosites in gp120 that had been digested by trypsin or trypsin/Glu-C. **(C)** Different types and relative ratios of O-glycans on each unambiguously (red oval) or ambiguously (yellow oval) assigned O-glycosites of the recombinant HIV-1 gp120 protein expressed in human cells.

By combining all the data from different digestion methods (trypsin and trypsin/Glu-C) and different mass spectrometry methods (sceHCD-MS/MS and EThcD-sceHCD-MS/MS), 26 potential O-glycosites were identified (**Supplementary Table S1**). Among them, nine potential O-glycosites were identified by both sceHCD-MS/MS and EThcD-sceHCD-MS/MS (**Figure 7A**), and ten potential O-glycosites were identified from both trypsin-and trypsin/Glu-C-digested intact O-glycopeptides (**Figure 7B**). In addition, we manually analyzed the spectra of these intact O-glycopeptides. Six O-glycosites could be assigned unambiguously, and twenty O-glycosites could be ambiguously assigned (**Figure 7C**). Among them, two O-glycosites (T60 and T468) could be identified by sceHCD-MS/MS because of the presence of only one potential O-glycosite in one intact O-glycopeptide of gp120 (**Supplementary Figure S5**). In contrast, five O-glycosites (S132, S308, T382, T419, and T468) could be confidently identified by EThcD-sceHCD-MS/MS because EThcD-sceHCD can provide more ion information for O-glycosites in an intact O-glycopeptide with multiple serine and/or threonine residues (**Supplementary Figure S6**). T468 was the most highly modified by five O-glycans: HexNAc(1)Hex(1)NeuAc(2) (45%), HexNAc(1)Hex(1)NeuAc(1) (43%), HexNAc(1) (7%), HexNAc(1)Hex(1) (4%), and HexNAc(2) (1%) (**Figure 7C**). These O-glycans containing sialic acid are the main component of the gp120 subunit. The biological function of these O-glycans at each site in gp120 requires further exploration.

Because of the considerable clinical need for the rational design of vaccines and targeted drugs, the analysis of glycans, glycosites, glycosidase-treated glycopeptides, and glycoproteins of recombinant gp120 has been reported in several studies (28). However, the analysis of intact N/O-glycopeptides with site-specific glycan information provides comprehensive and highly accurate information on a highly glycosylated protein containing many potential glycosites. In this study, we performed a simultaneous, in-depth, and comprehensive analysis of intact N/O-glycopeptides of recombinant HIV-1 gp120 using our well-established sequential glycoproteomic workflow based on sceHCD-MS/MS, EThcD-sceHCD-MS/MS, and Byonic software. Finally, 18 N-glycosites and 5 O-glycosites with attached glycans were assigned unambiguously by EThcD-sceHCD-MS/MS, and 14 N-glycosites and 2 O-glycosites with attached glycans were identified confidently by sceHCD-MS/MS. Our data indicated that EThcD-sceHCD-MS/MS was generally more reliable at generating fragment ion types suitable for robust characterization of intact N/O-glycopeptides than sceHCD-MS/MS. However, sceHCD-MS/MS outperformed EThcD-sceHCD-MS/MS in terms of the number of intact N-glycopeptides identified. Combining these methods can achieve better simultaneous analysis of the site-specific N/O-glycosylation of a highly glycosylated protein, such as viral glycoproteins, containing many potential glycosites in one process, gather more complete information, and reveal greater microheterogeneity details than either method alone. Sample preprocessing to produce as many intact N/O-glycopeptides with one potential glycosite as possible will assist in EThcD-sceHCD-MS/MS and sceHCD-MS/MS analysis because the spectral evidence from such glycopeptides is required for confident identification of N/O-glycosites. In addition, instrumentation, analytical methods, and data analysis software need to be adapted to develop intact N/O-glycopeptide analysis. A map of the site-specific N/O-glycosylation of gp120 would be a valuable resource for the rational design of vaccines and targeted drugs for HIV-1.

## Data Availability Statement

The datasets presented in this study can be found in online repositories. The names of the repository/repositories and accession number(s) can be found in the article/**Supplementary Material**.

## Author Contributions

HY, YC, MG, and JC designed research. YZ, SZ, and WaZ performed analyses of mass spectrometry data. YZ, YM, and WC adapted algorithms and software for data analysis. YZ, YL, LH, and WeZ coordinated acquisition, distribution and quality evaluation of samples. YZ and HY wrote the manuscript. All authors contributed to the article and approved the submitted version.

## Funding

This work was funded by grants from the National Natural Science Foundation of China (grant number 31901038), the China Postdoctoral Science Foundation (2019M653438, 2020M670063ZX), the Department of Science and Technology of Sichuan Province (2021YJ0479, 2020YFH0029), the Chengdu Science and Technology Department Foundation (2020-YF05-00240-SN), the National Clinical Research Center for Geriatrics, West China Hospital, Sichuan University (Z20201014), the 1.3.5 Project for Disciplines of Excellence, West China Hospital, Sichuan University (ZYGD18014).

## Conflict of Interest

The authors declare that the research was conducted in the absence of any commercial or financial relationships that could be construed as a potential conflict of interest.

## Publisher’s Note

All claims expressed in this article are solely those of the authors and do not necessarily represent those of their affiliated organizations, or those of the publisher, the editors and the reviewers. Any product that may be evaluated in this article, or claim that may be made by its manufacturer, is not guaranteed or endorsed by the publisher.

## Abbreviations

ACN, acetonitrile; AIDS, acquired immune deficiency syndrome; bNAbs, broadly neutralizing antibodies; DTT, dithiothreitol; Env, envelope; ESI, electrospray ionization; EThcD, electron transfer/higher-energy collisional dissociation; FA, formic acid; FASP, filter aided sample preparation; GPSMs, glycopeptide spectrum matches; HCD, higher-energy collisional dissociation; HILIC, hydrophilic interaction liquid chromatography; HIV-1, human immunodeficiency virus type 1; IAA, iodoacetamide; MS, mass spectrometry; NH_4_CO_3_, ammonium bicarbonate; PNGSs, potential N-linked glycosylation sites; PSMs, peptide spectrum matches; SARS-CoV, severe acute respiratory syndrome coronavirus; sceHCD, stepped collision energy/higher-energy collisional dissociation; TFA, trifluoroacetic acid; TRIS, tris(hydroxymethyl)aminomethane.
