# Supplementary material for: Sequential Analysis of the N/O-Glycosylation of Heavily Glycosylated HIV-1 gp120 Using EThcD-sceHCD-MS/MS
Source: Front Immunol. 2021 Oct 21;12:755568. doi: 10.3389/fimmu.2021.755568 (PMC8567067; doi:10.3389/fimmu.2021.755568)
Supplement: Supplementary file 1 [file DataSheet_1.docx]

**Supporting information for**

**Sequential analysis of the N/O-glycosylation of heavily glycosylated HIV-1 gp120 using EThcD-sceHCD-MS/MS**

Yong Zhang^1,4^, Shanshan Zheng^1^, Wanjun Zhao^2^, Yonghong Mao^3^, Wei Cao^1^, Wenjuan Zeng^1^, Yueqiu Liu^1^, Liqiang Hu^1^, Meng Gong^1,4^, Jingqiu Cheng^1^, Younan Chen^1*^, Hao Yang^1,4*^

^1^NHC Key Laboratory of Transplant Engineering and Immunology, Institutes for Systems Genetics; National Clinical Research Center for Geriatrics，West China Hospital, Sichuan University, Chengdu 610041, China.

^2^Department of Thoracic Surgery, West China Hospital, Sichuan University, Chengdu 610041, China.

^3^Institute of Thoracic Oncology, West China Hospital, Sichuan University, Chengdu 610041, China

^4^Sichuan Provincial Engineering Laboratory of Pathology in Clinical Application, West China Hospital, Sichuan University, Chengdu 610041, China.

***Corresponding Authors:** Hao Yang, PhD, Associate Professor, NHC Key Lab of Transplant Engineering and Immunology, West China Hospital, Sichuan University; Younan Chen, PhD, Associate Professor, NHC Key Lab of Transplant Engineering and Immunology, West China Hospital, Sichuan University

**Address:** No. 1, Keyuan 4th Road, Gaopeng Avenue, Hi-tech Zone, Chengdu 610041, China. Phone: +86-28-85164031; Fax: +86-28-85164031; E-mail: yanghao@scu.edu.cn and chenyounan@scu.edu.cn

**Supplementary Figures:**

**Figure S1.** Flow chart of parameter setting for EThcD-sceHCD-MS/MS method

**Figure S2.** Representative spectra of intact N-glycopeptides with unambiguously assigned N-glycosites by sceHCD-MS/MS.

**Figure S3.** Representative spectra of intact N-glycopeptides with unambiguously assigned N-glycosites by EThcD-sceHCD-MS/MS.

**Figure S4.** Comparison of the number of identified intact N-glycopeptides by different fragment methods

**Figure S5.** Representative spectra of intact O-glycopeptides with unambiguously assigned O-glycosites by sceHCD-MS/MS.

**Figure S6.** Representative spectra of intact O-glycopeptides with unambiguously assigned O-glycosites by EThcD-sceHCD-MS/MS.

**Figure S1.** Flow chart of parameter setting for EThcD-sceHCD-MS/MS method


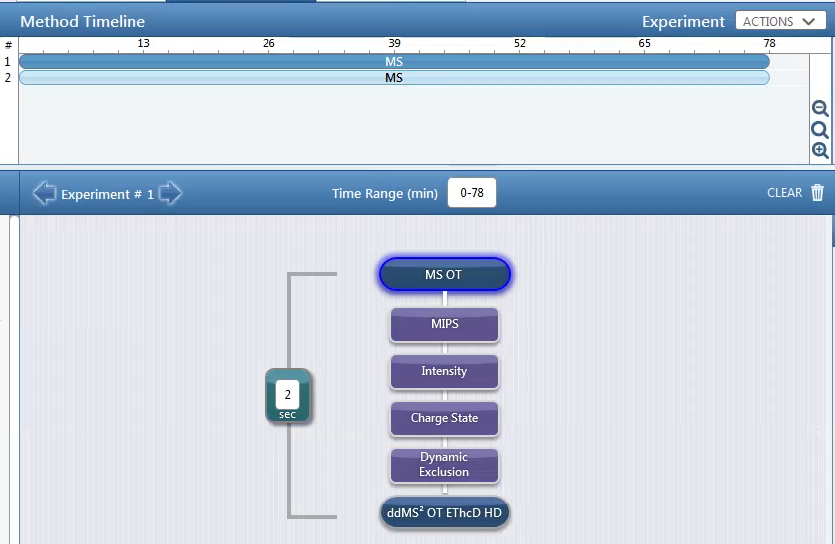

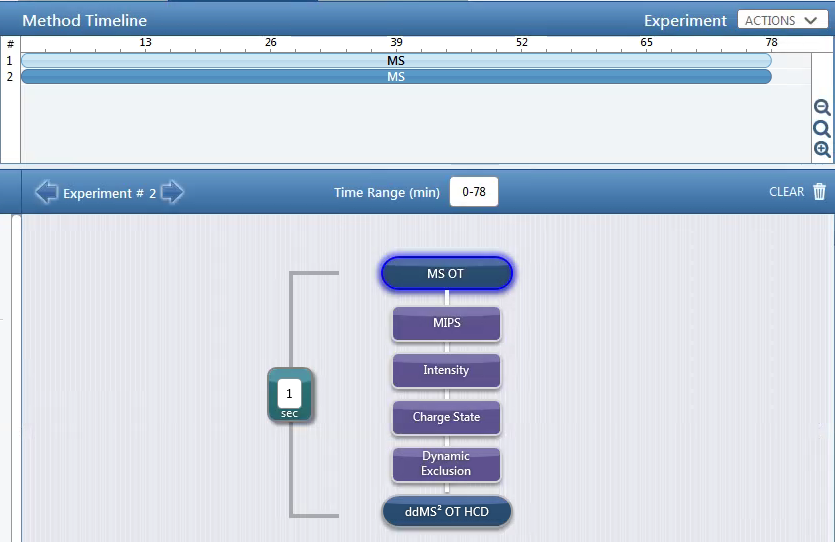


**Duty cyle 1 (EThcD)**

**Duty cyle 2 (sceHCD)**

**Figure S2.** Representative spectra of intact N-glycopeptides with unambiguously assigned N-glycosites by sceHCD-MS/MS.

**N58**

**
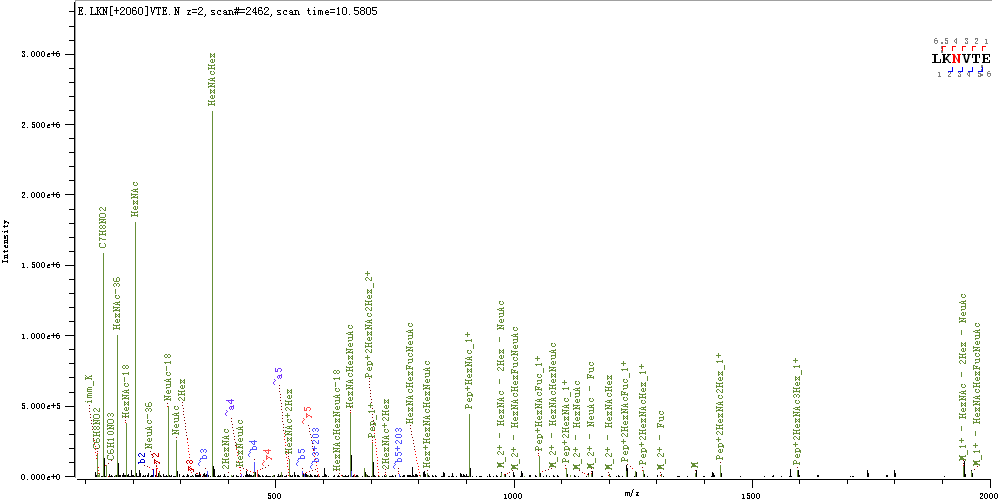
**

**N100**

**
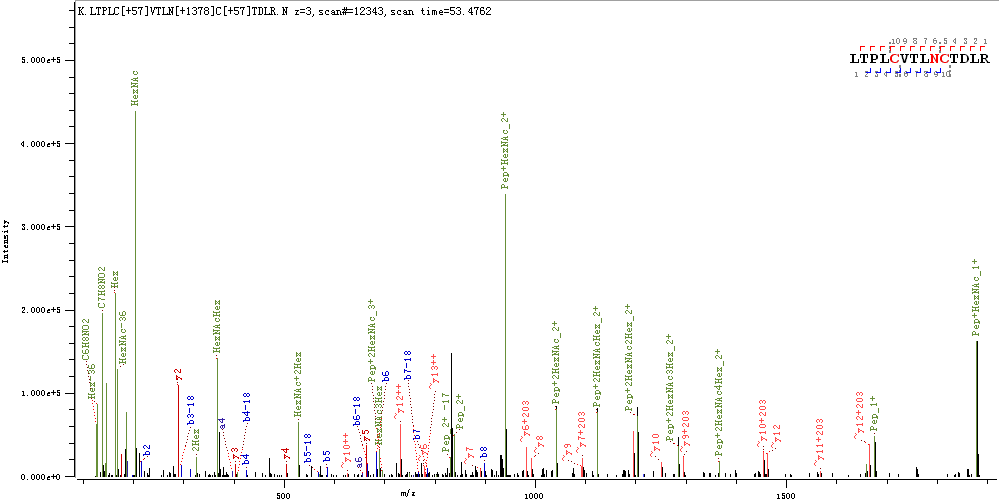
**

**N160**

**
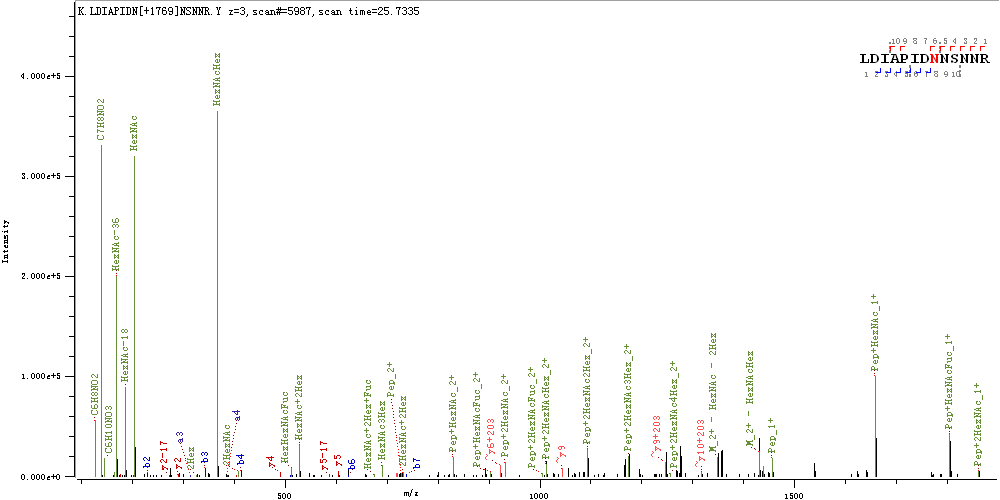
**

**N172**

**
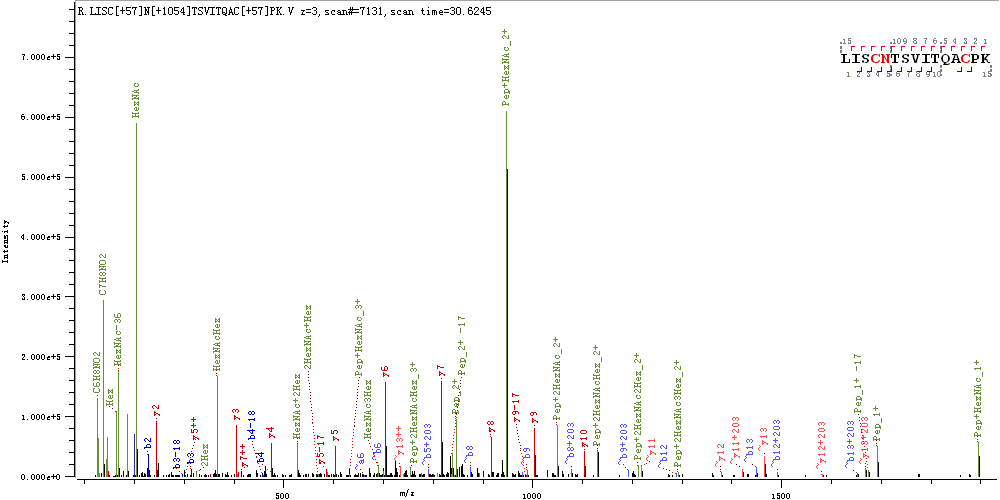
**

**N216**

**
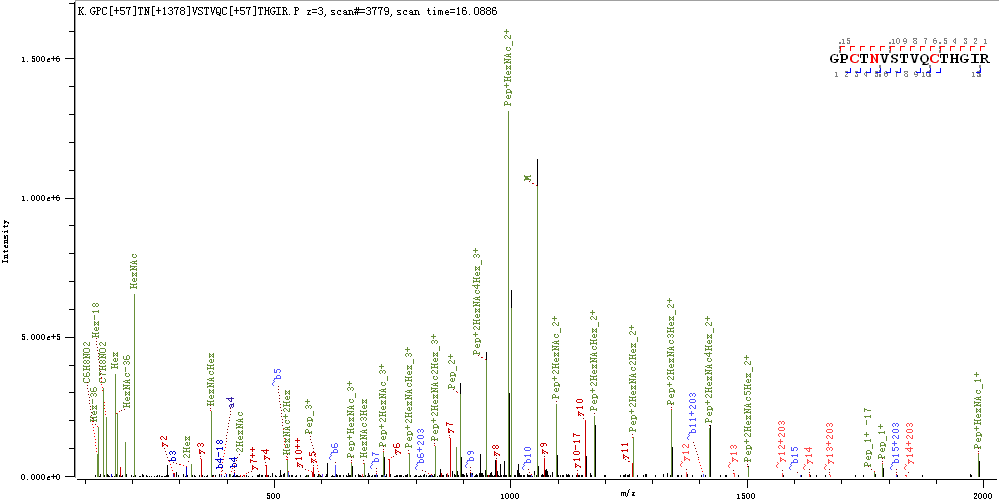
**

**N237**

**
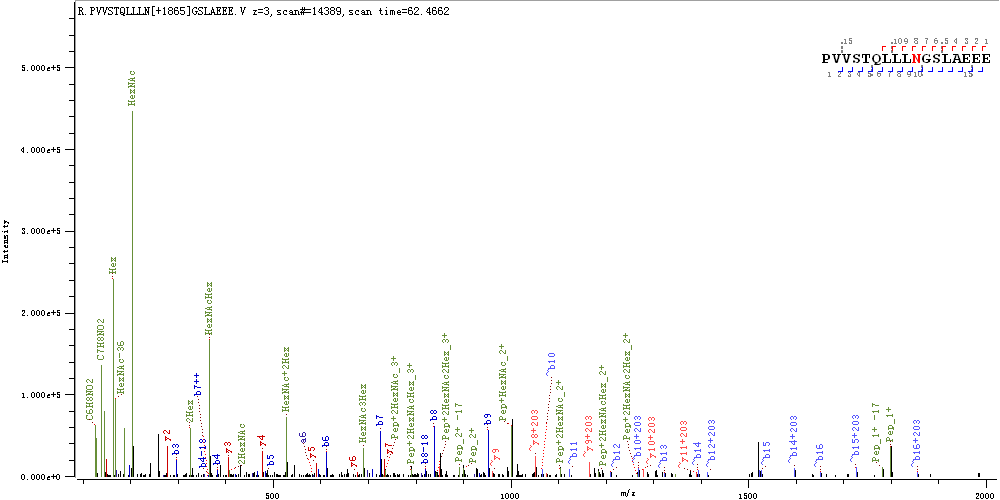
**

**N264**

**
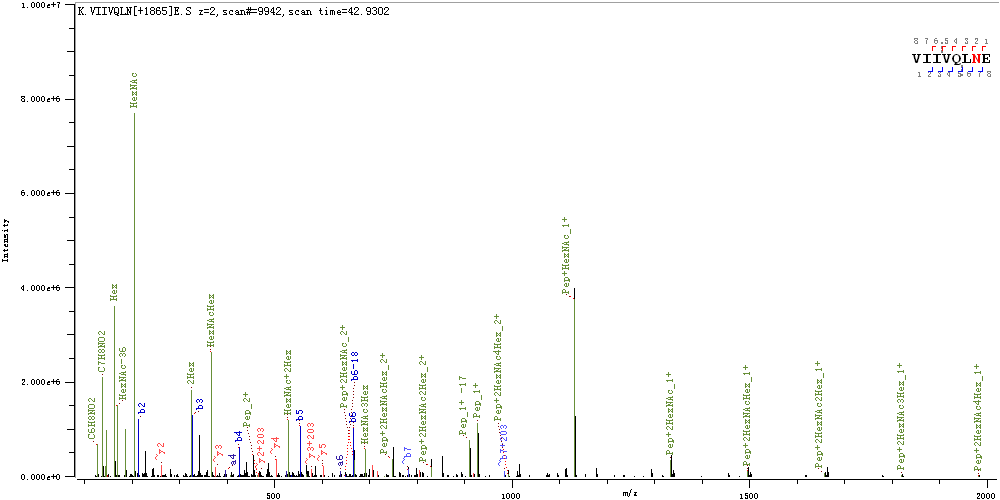
**

**N306**

**
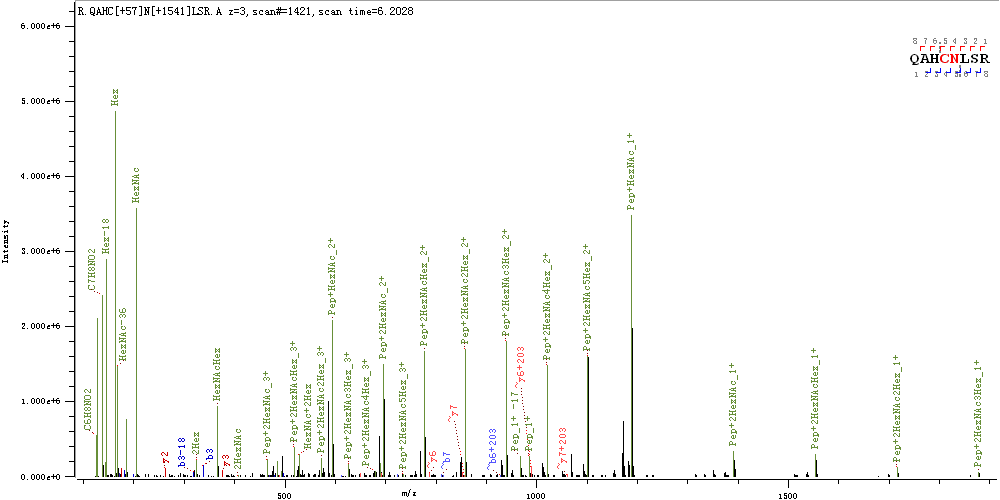
**

**N313**

**
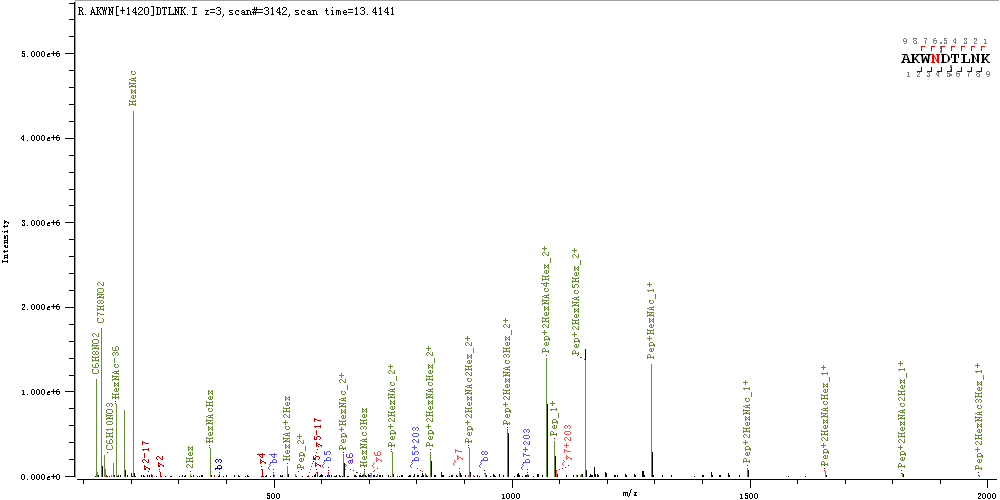
**

**N329**

**
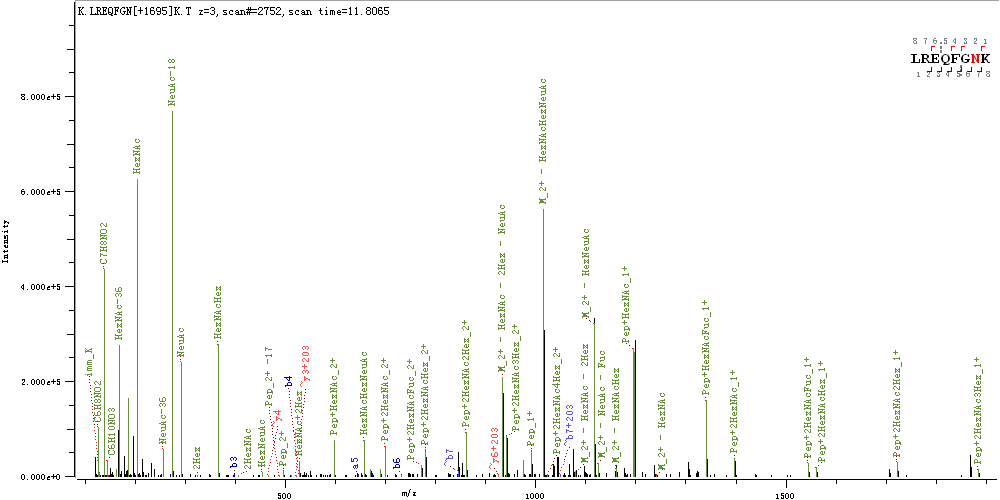
**

**N375**

**
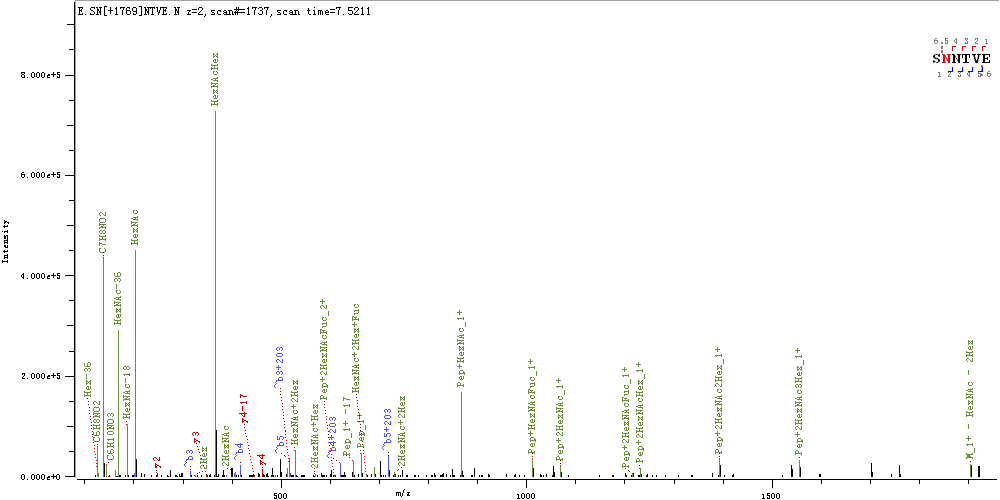
**

**N380**

**
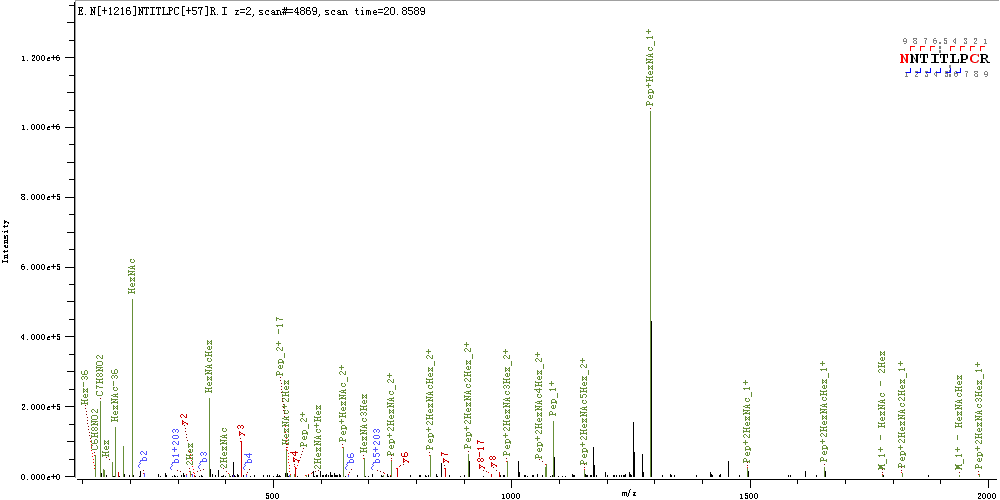
**

**M417**

**
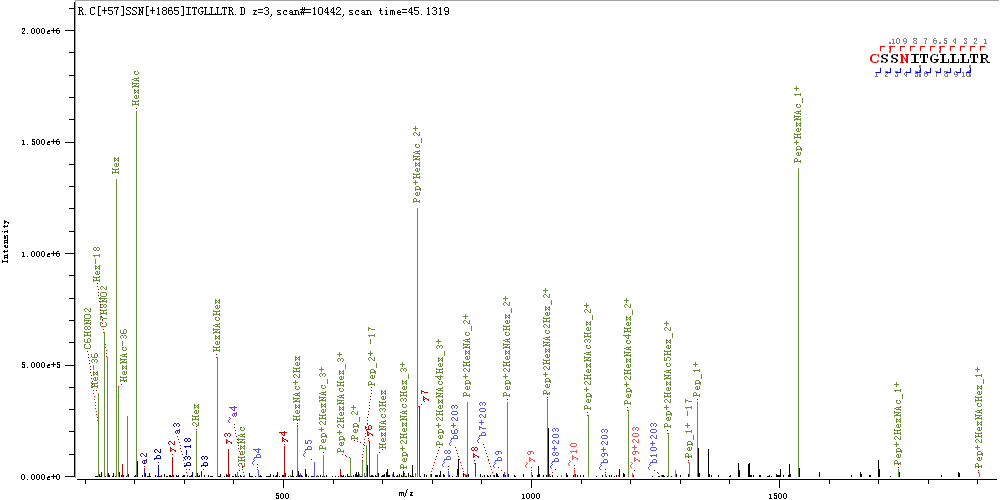
**

**N432**

**
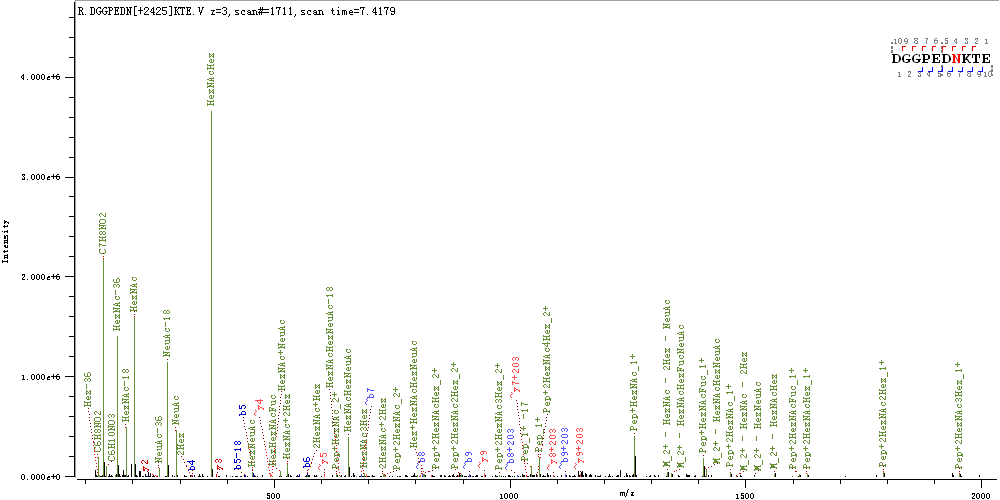
**

**Figure S3.** Representative spectra of intact N-glycopeptides with unambiguously assigned N-glycosites by EThcD-sceHCD-MS/MS.

**N58**

**
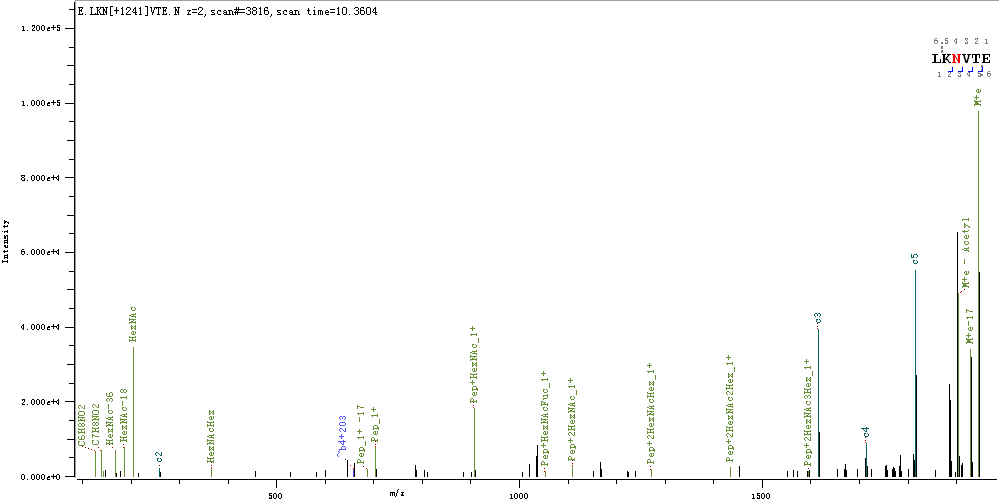
**

**N100**

**
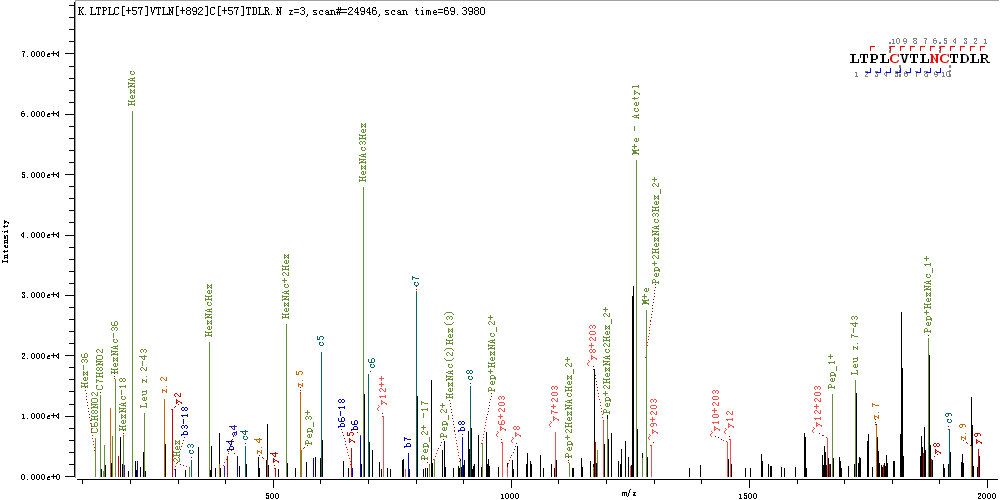
**

**N130 & N134**

**
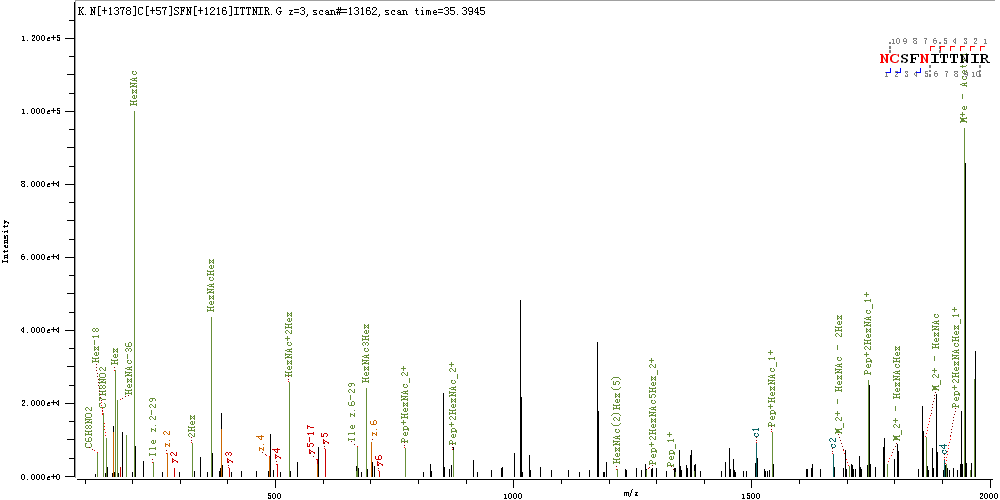
**

**N160**

**
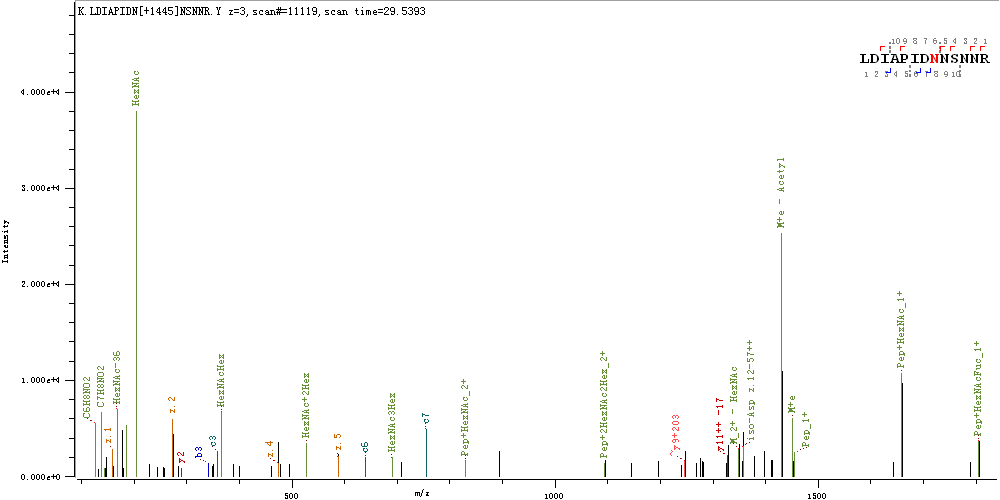
**

**N172**

**
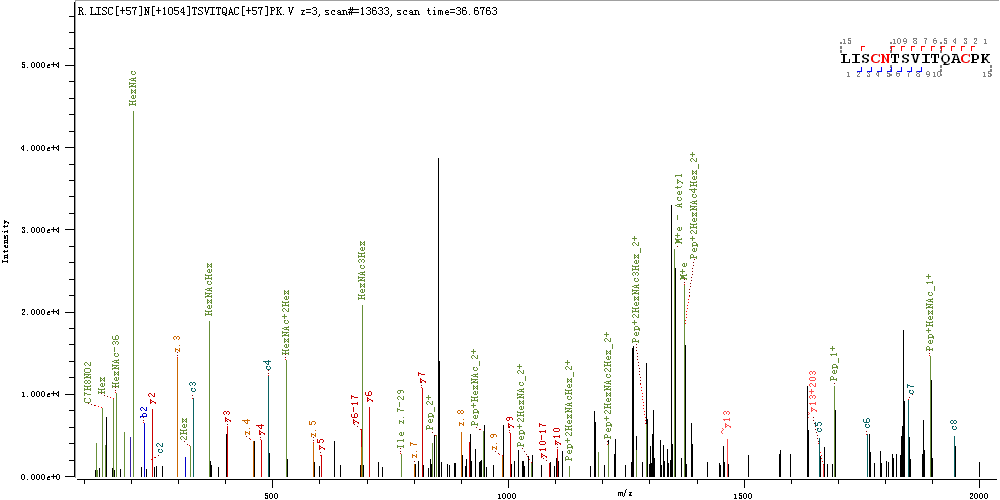
**

**N216**

**
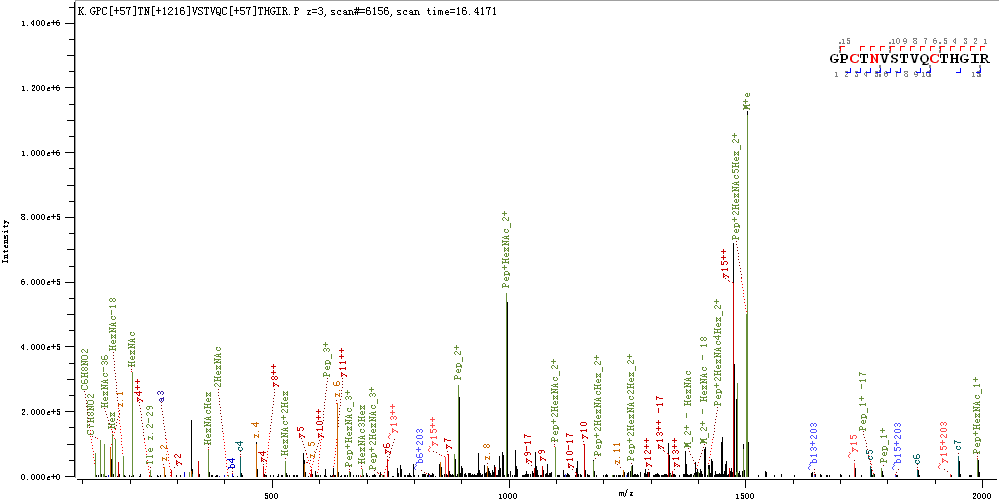
**

**N237**

**
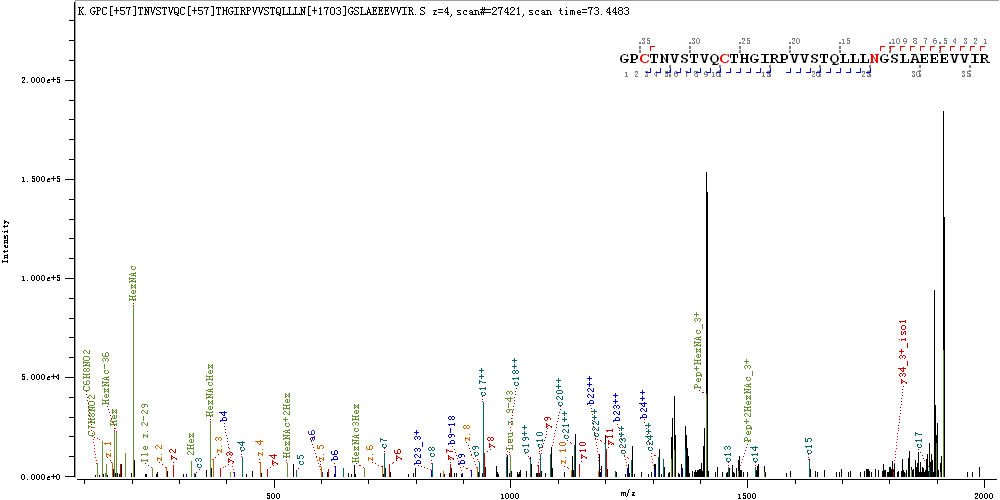
**

**N264**

**
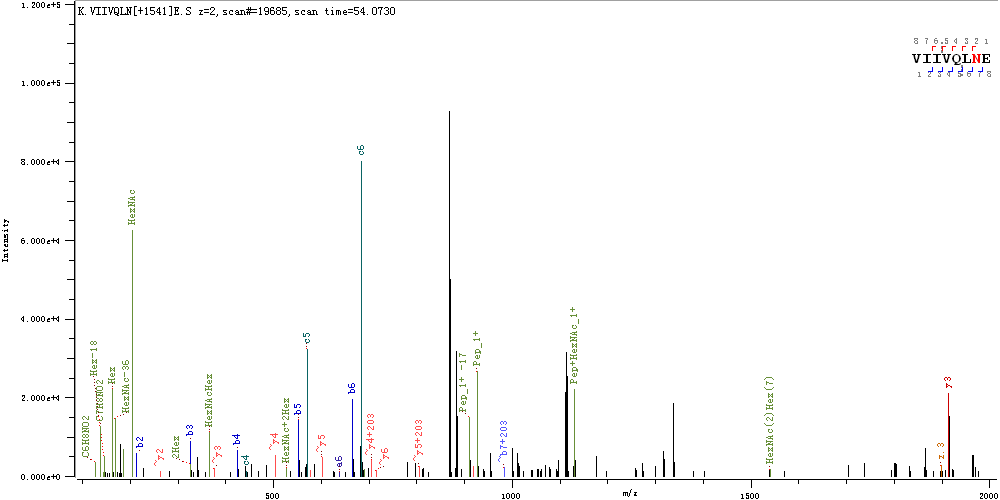
**

**N270 & N276**

**
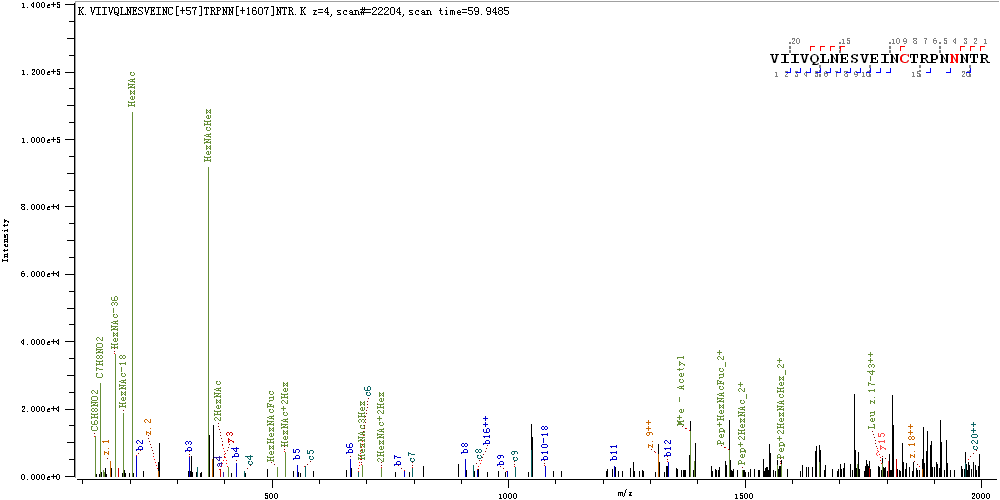
**

**N306**

**
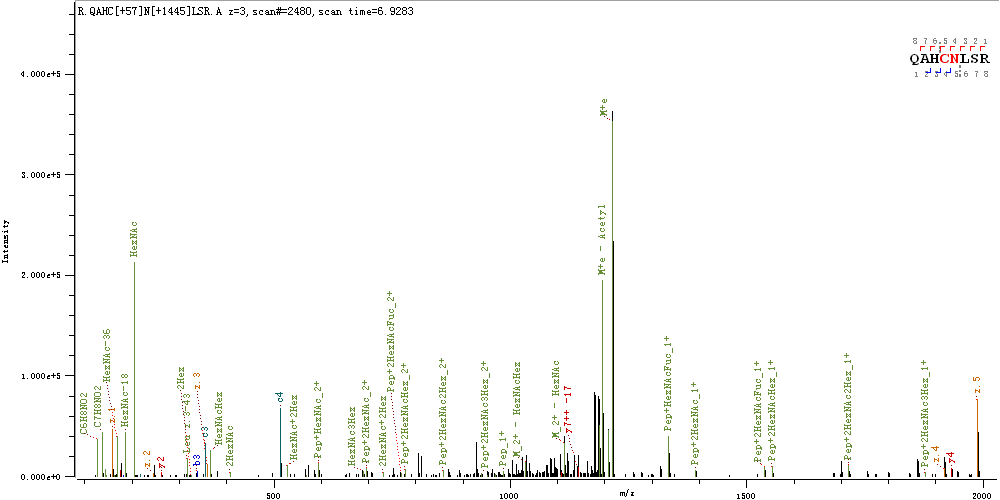
**

**N313**

**
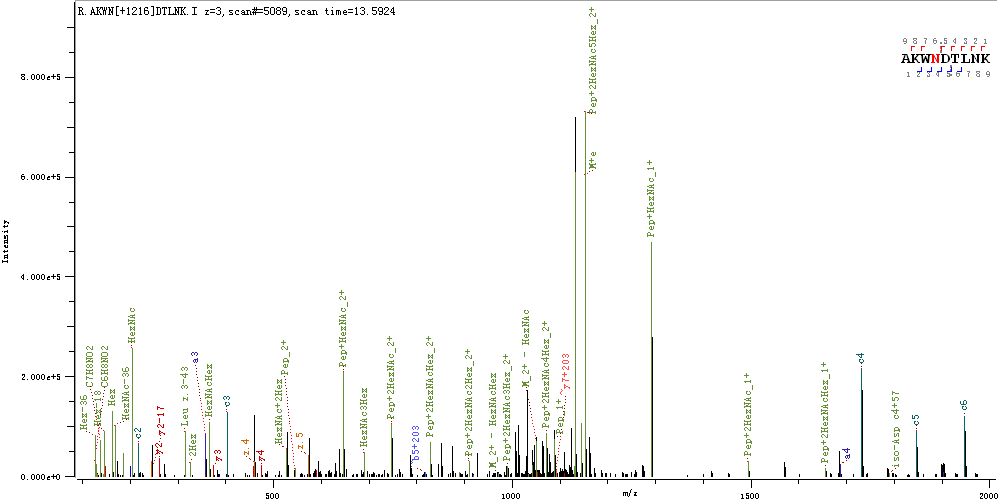
**

**N329**

**
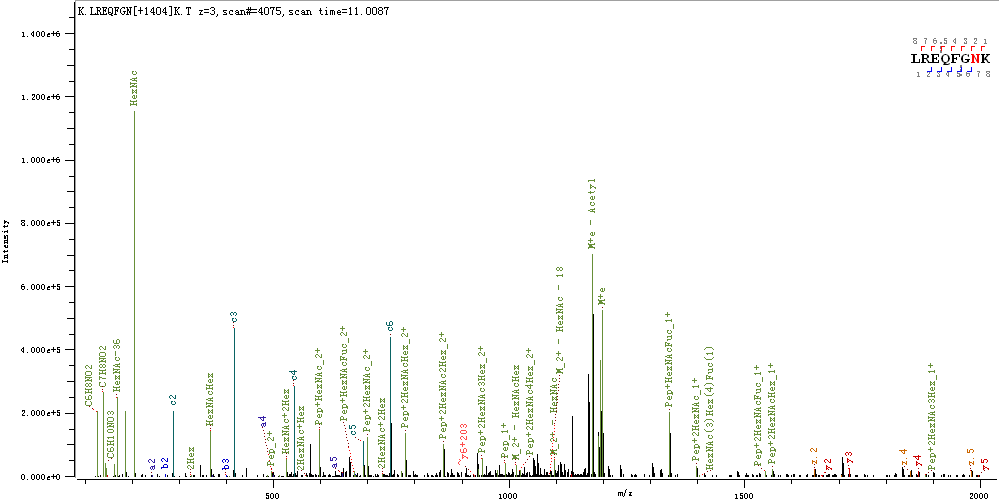
**

**N375**

**
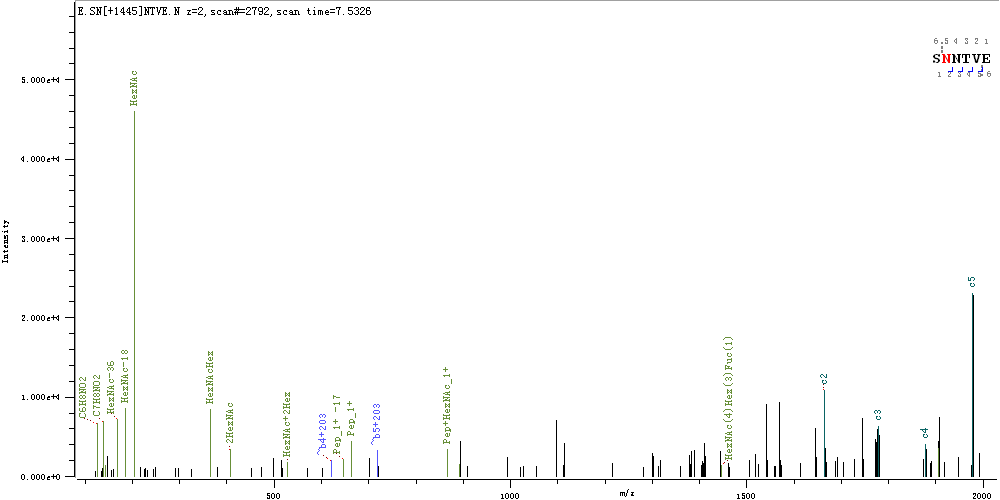
**

**N380**

**
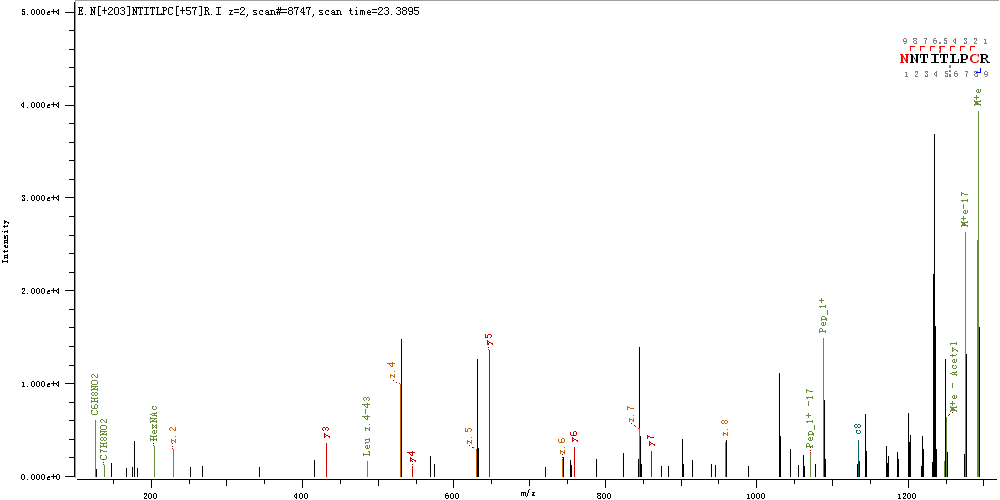
**

**M417**

**
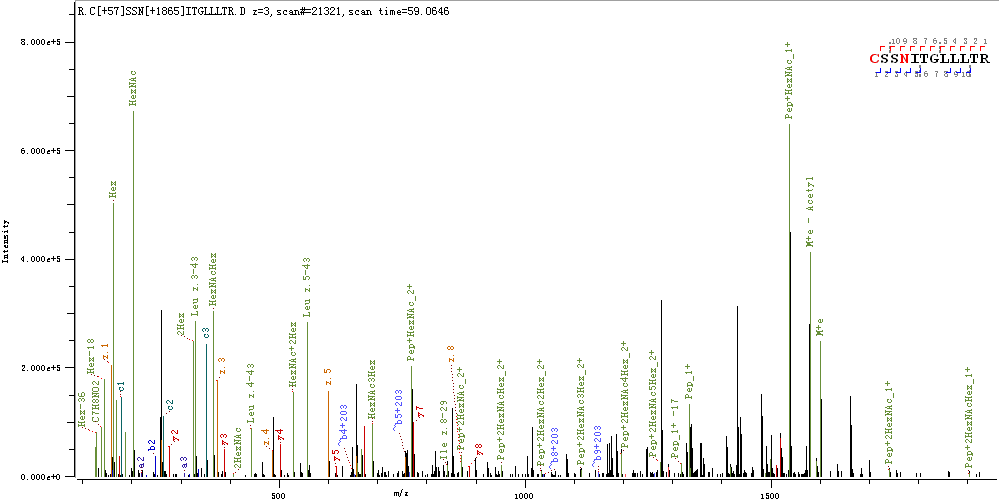
**

**N432**

**
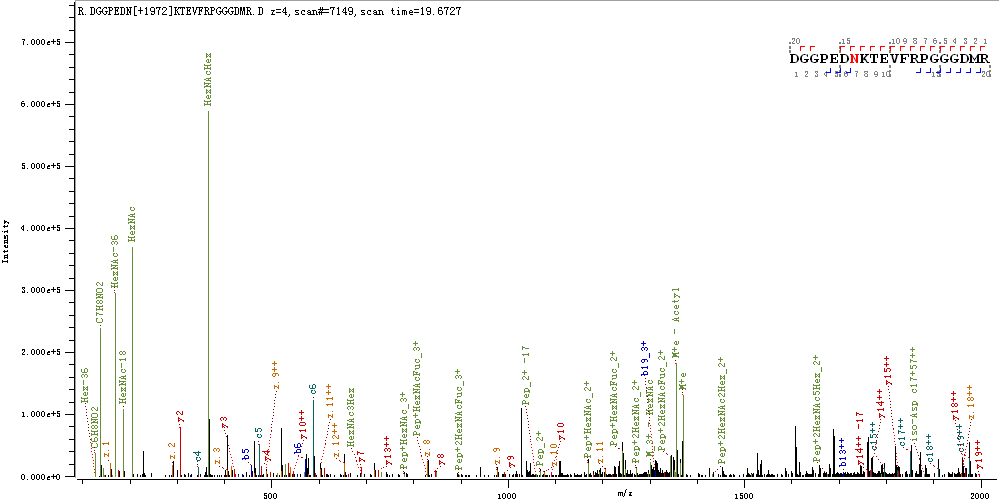
**

**Figure S4.** Comparison of the number of identified intact N-glycopeptides by different fragment methods


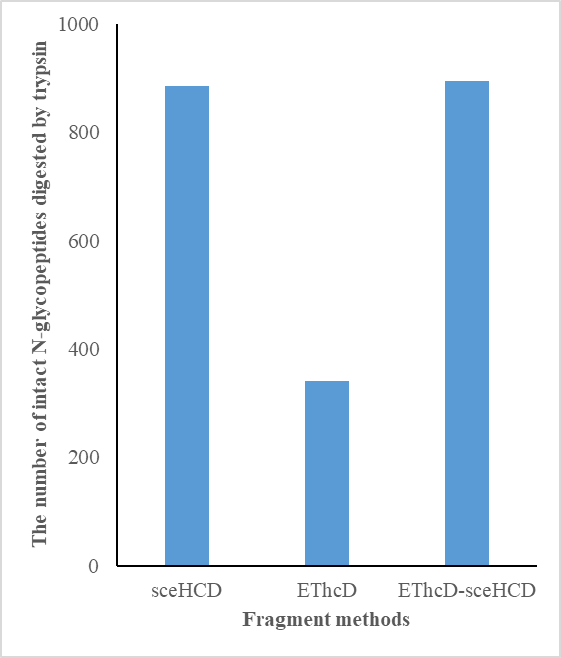


**Figure S5.** Representative spectra of intact O-glycopeptides with unambiguously assigned O-glycosites by sceHCD-MS/MS.

**T60**

**
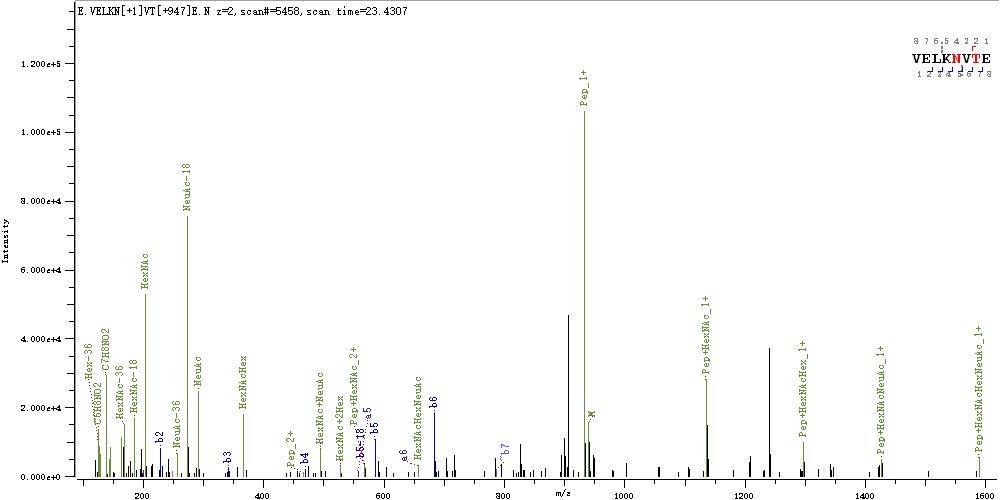
**

**T468**

**
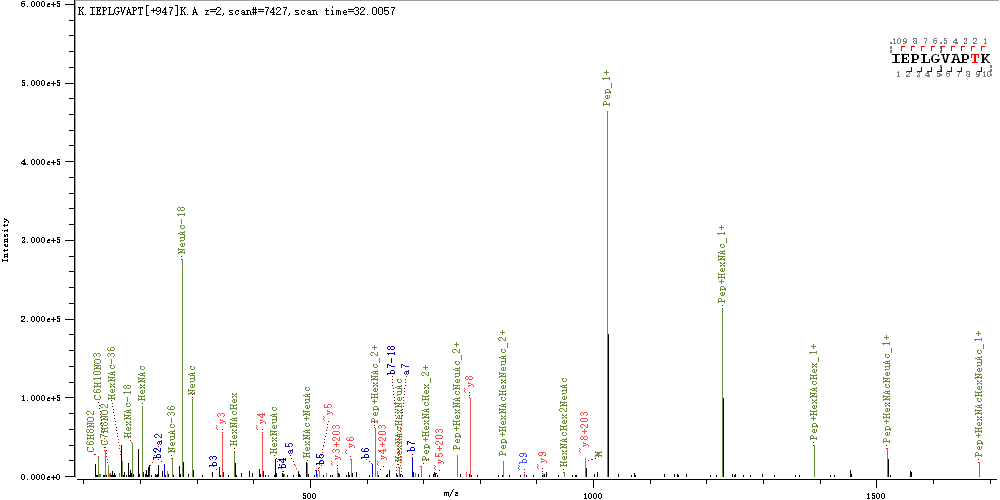
**

**Figure S6.** Representative spectra of intact O-glycopeptides with unambiguously assigned O-glycosites by EThcD-sceHCD-MS/MS.

**S132**

**
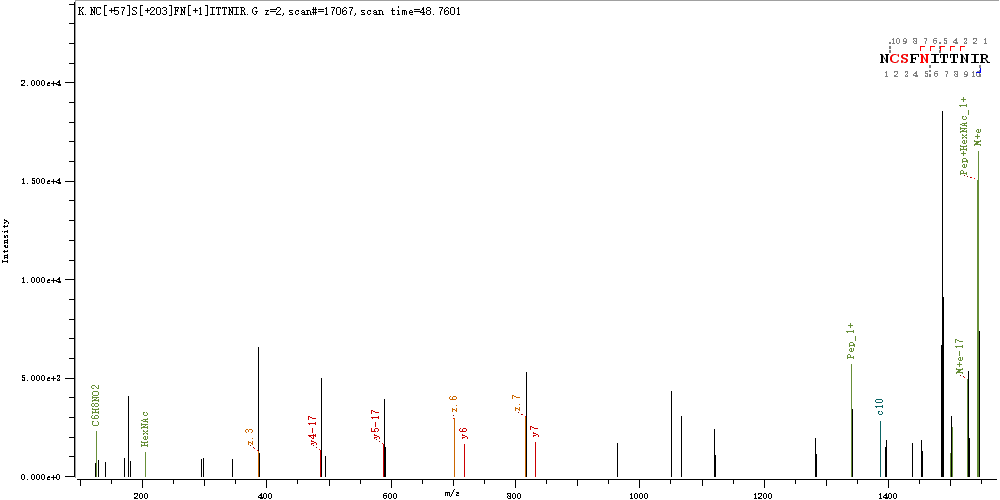
**

**S308**

**
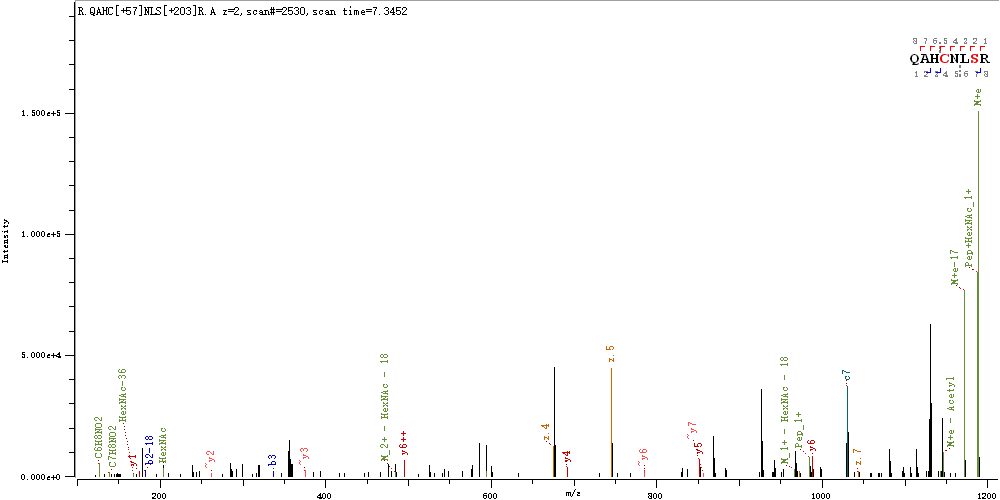
**

**T382**

**
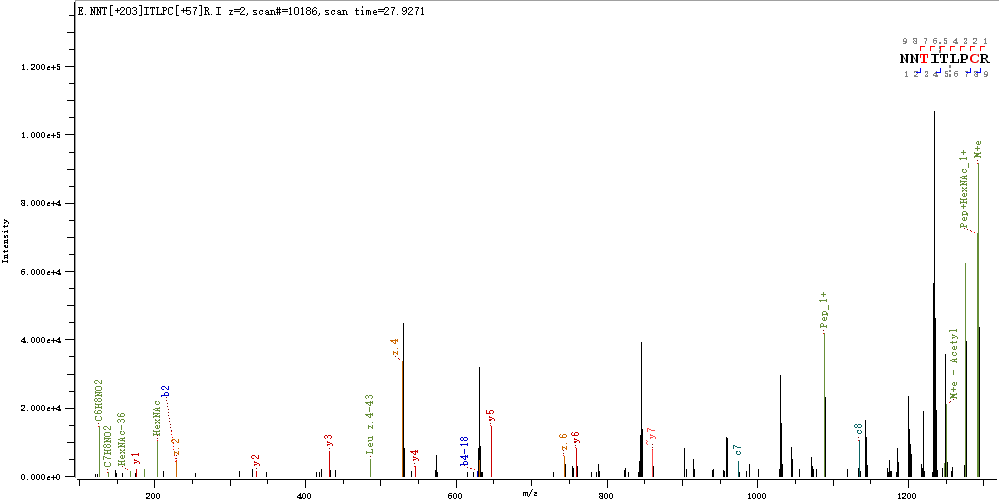
**

**T419**

**
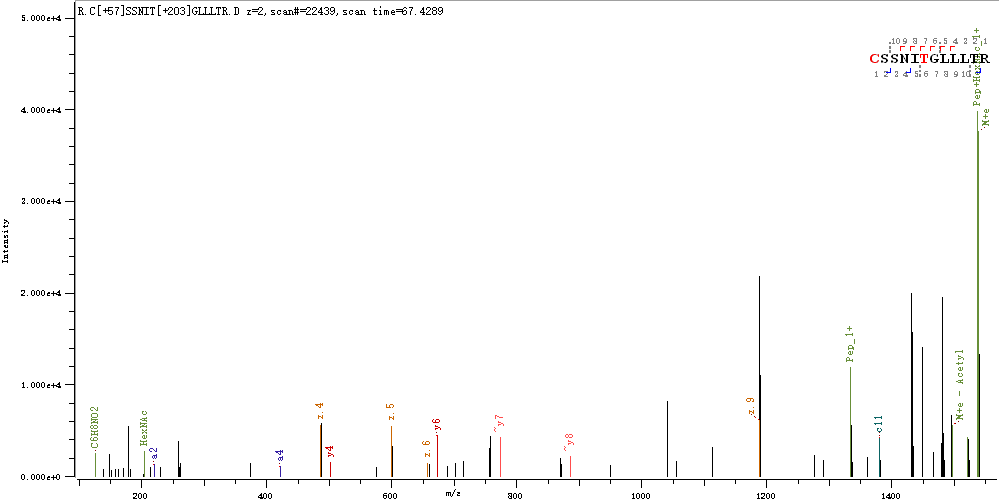
**

**T468**

**
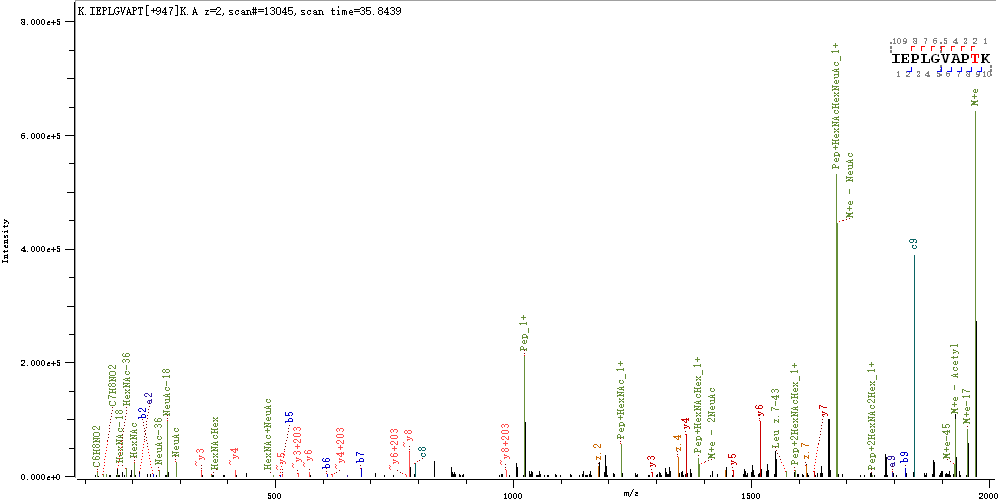
**
